# Molecular Changes In Cardiac Tissue As A New Marker To Predict Cardiac Dysfunction Induced By Radiotherapy

**DOI:** 10.3389/fonc.2022.945521

**Published:** 2022-07-26

**Authors:** Sónia Ribeiro, Ana Rita Simões, Filipe Rocha, Inês Sofia Vala, Ana Teresa Pinto, Augusto Ministro, Esmeralda Poli, Isabel Maria Diegues, Filomena Pina, Mohamed Amine Benadjaoud, Stephane Flamant, Radia Tamarat, Hugo Osório, Diogo Pais, Diogo Casal, Fausto José Pinto, Rune Matthiesen, Manuela Fiuza, Susana Constantino Rosa Santos

**Affiliations:** ^1^ Centro Cardiovascular da Universidade de Lisboa, Lisbon School of Medicine of the Universidade de Lisboa, Lisbon, Portugal; ^2^ Santa Maria University Hospital, Centro Hospitalar Universitário Lisboa Norte (CHULN), Lisbon, Portugal; ^3^ Department of Radiobiology and Regenerative Medicine, Institut de Radioprotection et de Sûreté Nucléaire, Fontenay-aux-Roses, France; ^4^ i3S-Instituto de Investigação e Inovação em Saúde, Universidade do Porto, Porto, Portugal; ^5^ Ipatimup-Institute of Molecular Pathology and Immunology of the University of Porto, Porto, Portugal; ^6^ Department of Pathology, Faculty of Medicine, University of Porto, Porto, Portugal; ^7^ NOVA Medical School, Universidade NOVA de Lisboa, Lisbon, Portugal; ^8^ Chronic Diseases Research Centre, NOVA Medical School, Faculdade de Ciências Médicas, Universidade NOVA de Lisboa, Lisbon, Portugal

**Keywords:** cardiotoxicity, radiotherapy, cardiac dysfunction, cardiac muscle, microvasculature, global longitudinal strain (GLS)

## Abstract

The contribution of radiotherapy, *per se*, to late cardiotoxicity remains controversial. To clarify its impact on the development of early cardiac dysfunction, we developed an experimental model in which the hearts of rats were exposed, in a fractionated plan, to clinically relevant doses of ionizing radiation for oncological patients that undergo thoracic radiotherapy. Rat hearts were exposed to daily doses of 0.04, 0.3, and 1.2 Gy for 23 days, achieving cumulative doses of 0.92, 6.9, and 27.6 Gy, respectively. We demonstrate that myocardial deformation, assessed by global longitudinal strain, was impaired (a relative percentage reduction of >15% from baseline) in a dose-dependent manner at 18 months. Moreover, by scanning electron microscopy, the microvascular density in the cardiac apex was significantly decreased exclusively at 27.6 Gy dosage. Before GLS impairment detection, several tools (qRT-PCR, mass spectrometry, and western blot) were used to assess molecular changes in the cardiac tissue. The number/expression of several genes, proteins, and KEGG pathways, related to inflammation, fibrosis, and cardiac muscle contraction, were differently expressed in the cardiac tissue according to the cumulative dose. Subclinical cardiac dysfunction occurs in a dose-dependent manner as detected by molecular changes in cardiac tissue, a predictor of the severity of global longitudinal strain impairment. Moreover, there was no dose threshold below which no myocardial deformation impairment was detected. Our findings i) contribute to developing new markers and exploring non-invasive magnetic resonance imaging to assess cardiac tissue changes as an early predictor of cardiac dysfunction; ii) should raise red flags, since there is no dose threshold below which no myocardial deformation impairment was detected and should be considered in radiation-based imaging and -guided therapeutic cardiac procedures; and iii) highlights the need for personalized clinical approaches.

## Introduction

Radiation therapy is an important tool for treating cancer. However, radiation-induced cardiovascular toxicity should be considered, mainly in diseases in which the thoracic region is irradiated. Technical progresses in radiation therapy improved cardiac sparing without affecting local tumor control and overall patient survival (1). These improvements contributed to the controversy about the impact of radiotherapy itself in cardiac toxicity, since radiation-induced cardiac disease most often occurs several years after radiotherapy (>10 years). For that reason, it is very difficult to distinguish its effects from previous chemotherapy and/or other risk factors such as smoking, hypertension, diabetes mellitus, coronary artery calcifications, and age. Recently, several studies have contributed to clarifying this aspect by identifying associations between cardiac substructure doses and cardiac outcomes (2–4) and/or survival and measuring regional dysfunction after cardiac radiation exposure (5, 6). Several imaging tools, such as echocardiography, cardiac magnetic resonance imaging, cardiovascular computed tomography (CT), and functional stress testing, are used to detect heart injury before clinical symptoms appear (7). Echocardiography is one of the first imaging tests used due to its wide availability and ability to assess structural changes and diastolic and systolic functions in a non-invasive way (8). Myocardial deformation parameters, and specifically the global longitudinal strain (GLS), measured by echocardiography, are important markers of early left ventricular (LV) subclinical dysfunction in breast cancer patients treated with radiotherapy, with a high prognostic value (8). It is more sensitive and robust than other conventional LV functional parameters, and its lowest levels have been associated with increased mortality. A relative percentage reduction of >15% from baseline is considered abnormal (8). Moreover, several circulating biomarkers were identified to detect subclinical cardiotoxicity (9) but recent studies have shown that they are not consistent biomarkers across different cancer types (10), highlighting the importance of future research to identify novel biomarkers independent of the cancer type. Despite improvements in radiation techniques and newer clinical studies revealing radiation-induced cardiac dysfunction, large gaps in the pathophysiological knowledge still exist and should be addressed towards a more personalized approach, earlier interventions, and update of guidelines.

Here, a translational experimental model was developed in which rat hearts were exposed for 23 days to daily doses of 0.04, 0.3, and 1.2 Gy, achieving cumulative doses of 0.92, 6.9, and 27.6 Gy, respectively. Using this model, we aimed to i) understand how different doses of ionizing radiation (IR) affect the cardiac tissue; ii) identify the early cardiac tissue molecules that, once affected, will play a role in the later development of subclinical cardiac dysfunction; and iii) understand whether there are doses less likely to induce cardiac dysfunction.

## Materials and methods

### Study Approval

All animal procedures were performed according to Directive 2010/63/EU. The procedures were approved by the institutional Animal Welfare Body, licensed by DGAV, the Portuguese competent authority for animal protection (license number 0421/000/000/2018).

### Rats and Reagents

Twelve to fourteen week-old female Wistar Han IGS (Crl : WI(Han) rats from Charles River Laboratories, Spain, were used. Animals were anaesthetized intraperitoneally with midazolam (4.76 mg/kg BW), medetomidine (0.356 mg/kg BW) and fentanyl (0.012 mg/kg BW). Sufficient sedation was verified by the absence of a paw withdrawal reflex. The anesthesia was reverted through subcutaneous injection using atipamezole (0.94 mg/kg BW) and flumazenil (0.2532 mg/kg BW). Rats were shaved in the thoracic region and anesthetized before the radiotherapy planning and echocardiogram. All animals were euthanized by exsanguination after deep anesthesia (2× dose above). A median xipho-pubic laparotomy was performed and the posterior vena cava and abdominal aorta were exposed. A catheter was inserted in the abdominal aorta into the descending thoracic aorta, followed by an incision in the vena cava to allow venous drainage. Perfusion was performed with physiological serum until the drainage fluid was clear. Rats were then injected with a glutaraldehyde solution or the heart was dissected (after confirmation of death).

### Irradiation Procedure

Animals were subjected to a CT simulation (Somatom Sensation, Siemens) consisting of helical CT scans with a 2-mm slice thickness and volumetric image reconstruction with an axial slice width of 1 mm. For a reproducible position, anesthetized rats were placed in an acrylic phantom in a supine position with fiducial markers (small radiopaque spheres) over the skin markers, allowing us to correlate their CT images with external anatomical references. The target structures (heart) and organs at risk (lungs and vertebral bodies) were delineated on the planned CT scan to virtually optimize the radiation beam delivery. A 3D computerized treatment planning system (XiO, Elekta), applying its superposition algorithm and heterogeneity correction, was used to calculate the radiation absorbed dose distribution throughout the rat body, generated by a 6 MV photon beam incident perpendicularly to the chest of the rat, with an equivalent square field size of 2.3 cm by 2.3 cm at 5 cm depth from the surface of the phantom. Due to the dimensions of the heart (about 1.45 cc), it was impossible to avoid irradiating the ipsilateral lung. Therefore, the volume irradiated encompassed the heart, the ipsilateral lung, and part of the contralateral lung.

Treatment plans were calculated for the prescribed doses of 0.04, 0.3, and 1.2 Gy. Finally, each anesthetized rat inside the phantom was set at the linear accelerator (LINAC) by aligning their fiducial markers with the LINAC light field and corresponding the room lasers with the isocenter of the beam. Rats were irradiated individually, according to the computerized treatment plan and the prescribed dose to the heart (0.04, 0.3, or 1.2 Gy), in an Elekta Synergy S LINAC, operating at a maximum dose rate of 600 MU per min. For each dose group (0.04, 0.3, and 1.2 Gy), the treatment was administered for 23 consecutive days (weekend excluded). Control rats were sham-irradiated (0.0 Gy) following the same procedure as the irradiated experimental groups.

### Echocardiography

An echocardiogram was performed as described (11). A Vivid T8 Ultrasound System (GE Medical Systems) was used with a 12 MHz sectorial probe. Images and videos were acquired throughout the parasternal and apical views. A pre-set was used to maintain image characteristics between specimens: frequency 5–10 MHz, depth 2.5 cm, frame-rate 124.7/s, Doppler sample 1.0 mm and color Doppler aliasing velocity of 40 cm/s. All exams were analyzed offline by two experienced cardiac sonographers using EchoPAC V202 (GE Medical Systems). To evaluate chamber dimensions, measurements were acquired in plain and apical views (11). To assess LV systolic function, ejection fraction was obtained using the volumetric modified Simpson method, cardiac output, cardiac index, mean S’-wave peak velocity, and mitral annulus plane systolic excursion (MAPSE) were studied (11). The right ventricle (RV) systolic function was studied by RV S’ velocity and tricuspid annulus plane systolic excursion (TAPSE). The diastolic function of LV was evaluated with the parameters obtained from LV inflow (E-wave, E/A ratio, and E-deceleration time) and spectral/pulsed tissue Doppler at the mitral annulus in the septal and lateral walls (E/E’ and E’/A’ ratios). Advanced measurements of myocardial deformation were performed using the software EchoPAC V202 (GE, Medical Systems), focusing on GLS by 2D speckle tracking using mean values obtained in mid-endocardial 4, 2, and 3 chambers views with aortic valve closure as reference.

### Tissue Digestion and Flow Cytometry

Heart digestion was performed according to a modified version of the protocol previously described (12). For example, heart tissue was minced and incubated with a digestion mix composed of basal DMEM (Gibco), 0.9 mg/ml of collagenase II (Invitrogen), and 0.25 U/ml of Dispase II (Roche), for 30 min at 37°C with agitation (200 rpm). Then, 7.5 μl/ml of DNAse (Roche) was added; after a short centrifugation (1,100 rpm), an equivalent volume of DMEM-10% of FBS (Gibco) was added to the supernatant. These steps were repeated twice. Cell suspension was centrifuged (5 min, at 1,100 rpm), and cells were treated with red blood cell lysis buffer (5 min, in the dark) and resuspended in flow cytometry solution (DMEM-2% FBS), filtered through a 70 μm cell strainer, and counted. For cellular senescence detection, 2 × 10^6^ cells were pre-treated with 100 nM bafilomycin A1 (Sigma-Aldrich), diluted in DMEM-10% FBS, at 37°C for 1 h, before adding C12FDG (ThermoFisher Scientific) to a final concentration of 33 μM, for an additional 2 h. For surface staining, cells were Fc-blocked with mouse anti-rat CD32 (D34-485, BD Biosciences), followed by incubation with antibody mix for 1 h, in the dark, at 4°C. Fluorescence-conjugated antibodies (BD Biosciences) for CD3 (1F4, BV421), CD90 (OX-7, BV650), CD45R (HIS24, BV786), CD31 (TLD3-A12, PE), CD11b/c (OX-42, PE-Cy7), and CD45 (OX-1, APC-Cy7) were used. Dilutions of 1:50 and 1:100 in flow cytometry solution were used for anti-CD3/anti-CD45 and for the remaining antibodies, respectively. Cells were then incubated with Zombie Aqua Dye (BioLegend) diluted 1:500 in PBS 1×, for 20 min at room temperature. Before flow cytometer acquisition (Fortessa X-20, BD Biosciences), cells were filtered using 70 μm strainers. To define negative populations per marker, the respective fluorescence minus one control was used. Data were analyzed using FlowJo v10 software (BD Biosciences).

### SEM of Vascular Corrosion Casts

After perfusion, animals were injected with 2.5% glutaraldehyde (Sigma-Aldrich) through the abdominal aorta. MercoxÒ CL-2R (Japan Vilene Co) was prepared by mixing the base resin with 2.5% benzoyl peroxide catalyst and then injected through the aorta. After overnight at 40°C, heart was carefully dissected and corroded by rinsing in a 10% potassium hydroxide, until only the vascular casts of the coronary vessels remained. These were dried, placed in a SEM appropriate stab. Vascular casts were cathode sputtered 400 angstrom gold coating with a gold sputtering machine and examined using the scanning electron microscope JEOL model JSM-7001F, with acceleration voltage of 2-30 kV. The average vascular cast proportion in several successive 100x magnification SEM fields from apical to basal was assessed. For each apex image, a region of interest (ROI) was defined, and microvascular density was quantified in equivalent ROIs and density measurements performed using ImageJ software. "Line 318 "RNA Extraction, cDNA synthesis, and Quantitative Real-Time PCR.

### RNA Extraction, cDNA Synthesis, and Quantitative Real-Time PCR

Total RNA was extracted using an RNeasy Fibrous Micro Kit (QIAGEN) according to the instructions of the manufacturer. For the synthesis and preamplification of CDNA, the RT2 Nano PreAMP cDNA synthesis kit (QIAGEN) was used with three rounds of preamplification using the same genes described below. The mRNA expression levels were assessed by qRT-PCR using a Power SYBR^®^ Green system (Invitrogen) on a 7500/7500 Fast Real-Time PCR System (Applied Biosystems). The housekeeping gene was 18S. The initial denaturation step was 10 min at 95°C, followed by 50 cycles of 15 s at 95°C, and 1 min at 60°C.

The relative quantification was performed through the 2^(−Delta Ct) method, where Delta Ct = Ct (sample) − Ct (reference gene). Data for each animal are represented as a Log_2_ of fold change (2^(−Delta Ct)), relative to the fold change average of the 0.0 Gy group.

Gene-specific primers are detailed as follows (5’–3’):

*Il6_*F ACAGAAGGAGTGGTAAGGA
*Il6_*R CACACTAGGTTTGCCGAGTAG
*Vcam1_*F TCTTGGAGAACCCAGACAGA
*Vcam1_*R GGTAAGAGTGCTCATCCTCAAC
*Tnc_*F CAAGCCCTTGGCTGAAATTG
*Tnc_*R CGATGGAGTACTGGTTGTCTTC
*Ccl2_*F CAGAAACCAGCCAACTCTCA
*Ccl2_*R CTACAGGCAGCAACTGTGAA
*Sele_*F GCAGAATTCCTCCTGACTCTTC
*Sele_*R CTGGAACTAGCTCTTGACACAC
*Ffg2_*F CAAGCAGAAGAGAGAGGAGTTG
*Ffg2_*R CTCCAGGCGTTCAAAGAAGA
*Tgfb2_*F CAGATCCTGAGCAAGCTGAA
*Tgfb2_*R GTCCCTGGTACTGTTGTAGATG
*Tgfb1_*F GCAACAATTCCTGGCGTTAC
*Tgfb1_*R GTATTCCGTCTCCTTGGTTCAG
*Ccn2_*F ACCTGTGCCTGCCATTAC
*Ccn2_*R TCCCTTACTCCCTGGCTTTA
*Col1a1_*F CCAATGGTGCTCCTGGTATT
*Col1a1_*R GTTCACCACTGTTGCCTTTG
*Fap*_F GGTCCTACGGAGGTTATGTTTC
*Fap_*R CCGTGTAGATAGAGGCGTAGTA
*Vim_*F CAGGAACAGCATGTCCAGAT
*Vim_*R TCTTGGCAGCCACACTTT
*Thy*1_F GTGTATGATGGGAGTGAAGAG
*Thy*1_R CTCTTTCAGGCATCTGGCTTAG
*Acta2*_F AGGGAGTGATGGTTGGAATG
*Acta2_*R GGTGATGATGCCGTGTTCTA
*Vegfa_*F GAAGACACAGTGGTGGAAGAAG
*Vegfa_*R ACAAGGTCCTCCTGAGCTATAC
*Kdr_*F TCCCAGAGTGGTTGGAAATG
*Kdr_*R ACTGACAGAGGCGATGAATG
*Sod2_*F TGAGGTCAGTAGGGTGTCTT
*Sod2_*R GGGAAAGGGTGTCCTTCTATTG
*18S*_F GCCCTATCAACTTTCGATGGTAGT
*18S*_R CCGGAATCGAACCCTGATT

### Protein Extraction and Digestion

Heart tissue proteins were extracted and solubilized with 100 mM Tris pH 8.5, 1% sodium deoxycholate, 10 mM tris(2-carboxyethyl)phosphine (TCEP), 40 mM chloroacetamide, and protease inhibitors for 10 min at 95°C and 1,000 rpm. Protein extract was sonicated for 10 cycles, 30 s on/30 s off (Bioruptor Plus, Diagenode). After centrifugation at 13,200 rpm for 5 min, 100 μg of protein were processed for proteomics analysis following the solid-phase-enhanced sample-preparation (SP3) protocol (13). Enzymatic digestion was performed using Trypsin/LysC (2 μg) overnight at 37°C and 1,000 rpm. The resulting peptides were cleaned-up and desalted with C18 microcolumns and further quantified.

### NanoLC-MS/MS

Protein identification was performed by nanoLC-MS/MS. This equipment is composed of an Ultimate 3000 liquid chromatography system coupled to a Q-Exactive Hybrid Quadrupole-Orbitrap mass spectrometer (Thermo Scientific). Approximately 500 ng of peptides were loaded onto a trapping cartridge (Acclaim PepMap C18 100 Å, 5 mm × 300 µm i.d., 160454, Thermo Scientific) in a mobile phase of 2% ACN, 0.1% FA at 10 µl/min. After 3 min loading, the trap column was switched in-line to a 50 cm by 75 μm inner diameter EASY-Spray column (ES803, PepMap RSLC, C18, 2 μm, Thermo Scientific) at 300 nl/min. Separation was generated by mixing A: 0.1% FA and B: 80% ACN with the following gradient: 5 min (2.5% B to 10% B), 120 min (10% B to 30% B), 20 min (30% B to 50% B), 5 min (50% B to 99% B), and 10 min (hold 99% B). Subsequently, the column was equilibrated with 2.5% B for 17 min. Data acquisition was controlled by Xcalibur 4.0 and Tune 2.9 software (Thermo Scientific). The mass spectrometer was operated in data-dependent (dd) positive acquisition mode, alternating between a full scan (m/z 380–1,580) and subsequent HCD MS/MS of the 10 most intense peaks from the full scan (normalized collision energy of 27%). The ESI spray voltage was 1.9 kV. Global settings: lock masses best (m/z 445.12003), lock mass injection Full MS, chrom. peak width (FWHM) of 15 s. Full scan settings: 70 k resolution (m/z 200), AGC target of 3 × 10^6^, maximum injection time of 120 ms. dd settings: minimum AGC target of 8 × 10^3^, intensity threshold of 7.3 × 10^4^, charge exclusion: unassigned, 1, 8, >8, peptide match preferred, exclude isotopes on, dynamic exclusion of 45 s. MS2 settings: microscans 1, resolution of 35 k (m/z 200), AGC target of 2 × 10^5^, maximum injection time of 110 ms, isolation window of 2.0 m/z, isolation offset of 0.0 m/z, spectrum data type profile.

### Protein Identification and Quantification

Protein identification and quantitative analysis was performed by Proteome Discoverer™ (Thermo Scientific), MaxQuant (14) and VEMS (15) considering the information from the UniProt protein database for *Rattus norvegicus* taxonomic selection. Quantitative analysis was compared and merged to create a consensus quantitative report to minimize computational artifacts. Quantitation was performed at the peptide and protein level using both ion and spectral count-based methodologies.

### Database Dependent Search of MS Data

The mass accuracy was set to 5 ppm on the peptide level and 0.01 Da on the fragment ions. A total of four missed cleavages were used. Carbamidomethyl was set as a fixed modification. Methionine oxidation, N-terminal protein acetylation, di-glycine tag on lysine, lysine acetylation, tyrosine nitration, tyrosine phosphorylation, threonine phosphorylation, serine phosphorylation, N-terminal water loss, N-terminal NH_3_ loss, glutamine deamidation, and asparagine deamidation were set as variable modifications.

The MSMS data were searched against all reviewed rat proteins from UniProt (24 June 2019) with the concatenation of the sequences in reverse, maintaining only lysine and arginine in place. The data were searched and quantified with both MaxQuant and VEMS (14). For MaxQuant (15), the variable modifications such as methionine oxidation, N-terminal protein acetylation, glutamine deamidation, and asparagine deamidation were included.

### Quantitative Analysis of MS Data

Quantitative data from MaxQuant and VEMS were analyzed in the R statistical programming language. Protein iBAQ values from the two programs were preprocessed by three methods: 1) removing common MS contaminants followed by Log_2_ (x + 1) transformation, 2) removing common MS contaminants followed by Log_2_ (x + 1) transformation and quantile normalization, and 3) removing common MS contaminants followed by Log_2_ (x + 1) transformation, quantile normalization and abundance filtering to optimize the overall Gaussian distribution of the quantitative values. The distribution of the quantitative values in each sample for each processing method was analyzed. PCA was performed using the R statistical programming language. The three different preprocessing methods led to similar results, and therefore the quantitative data obtained using preprocessing method 2 was used for further analysis.

To define differentially expressed proteins, statistical analysis using the R package limma was used, and each irradiated experimental group was compared with the sham-irradiated group. Correction for multiple testing was applied using the described method (16).

### Functional Enrichment Analysis

Functional enrichment based on the hypergeometric probability test was used as described (17, 18). Functional enrichment was achieved by extracting all functional categories for which at least a sample showed significant enrichment based on the hypergeometric probability test. For these categories, the number of proteins matching the functional category was extracted and these were processed by multiple corrections of *P*-values (16). For the significant functional categories, −Log_10_ (*P*-values) of enrichment were represented in a heatmap for cellular components, biological processes, molecular function, and KEGG.

### Western Blot

Cardiac lysates (25–50 mg) previously prepared were resolved on SDS-PAGE gels and transferred to nitrocellulose membranes (Amersham). Immunoblot analysis was performed with the primary antibodies against Myosin6 (ab207926), Calsequestrin (ab3516), or cTnT (ab209813). After the respective secondary antibody binding, blots were developed with Pierce ECL Western Blotting substrate (Thermo Scientific). Densitometric quantitation of western blot images was performed using ImageJ^®^ open source software.

### ELISA

The cTnT concentration was measured in triplicate from rat blood plasma using the rat cTnT ELISA kit (Elabscience), according to the instructions of the manufacturer.

### Statistical Analysis

Experimental data are presented as mean ± standard deviation (SD). Data were analyzed with GraphPad Prism 8 (GraphPad Software). Data normality was confirmed with the Shapiro–Wilk test. Significance of differences was assessed by one-way analysis of variance (ANOVA) followed by Bonferroni correction in the case of multiple groups if the distribution of the variable was normal; otherwise, Kruskal–Wallis tests were used employing the Dunn’s adjustment test. All probability values reported are two-sided, with *P <*0.05 considered significant. The number of experimental samples was the same except when: the values of expression were undetectable (qRT-PCR, ELISA), the amount of protein was not enough (western-blot) or the hearts were damaged during the sample processing of vascular casts (SEM).

## Results

### GLS is Significantly Decreased by 0.92, 6.9, and 27.6 Gy

**Figure 1:**
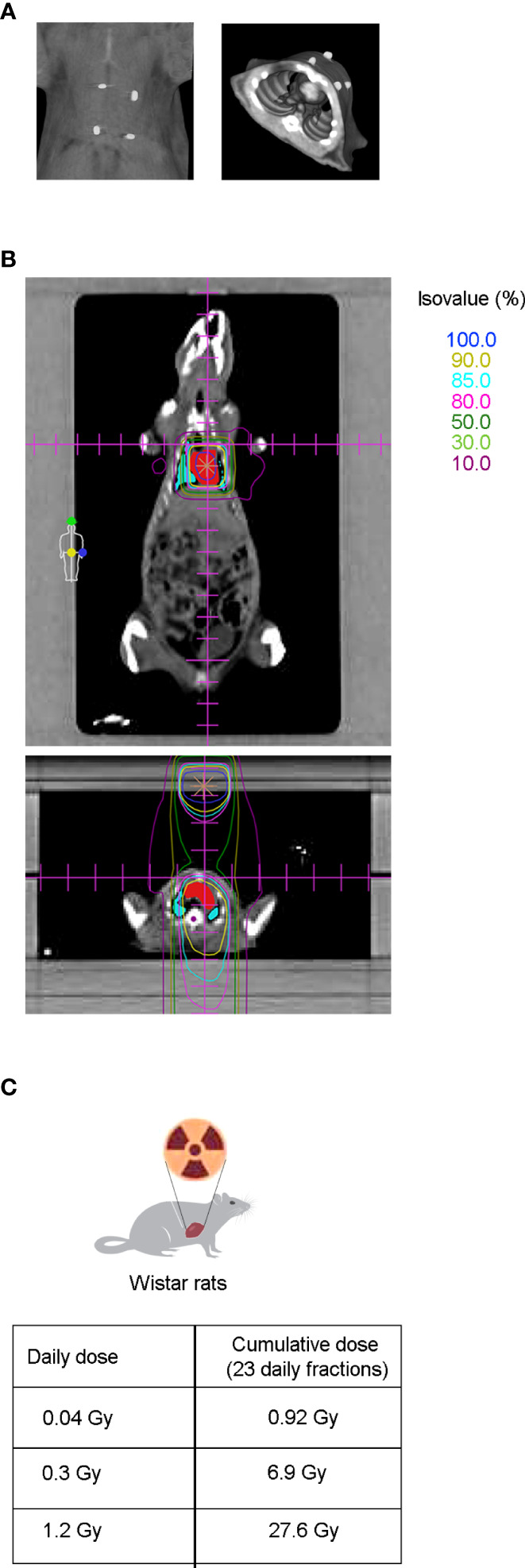
CT simulation and dose distribution planning preceded thoracic radiation therapy in Wistar rats. **(A)** After echocardiography, radiopaque fiducial markers were placed over the shaved skin of the animal (left panel) to correlate the CT images to the external anatomical references used during animal setup prior to RT (right panel). **(B)** Coronal (upper panel) and axial view (lower panel) from CT-based radiation therapy planning dose distribution. Isodose curves are shown in a range of 100% (dark blue), to 10% (dark pink), being the most internal ones: 90% (yellow), 85% (light blue) and 80% (light pink), for the prescribed doses of 0.04, 0.3, and 1.2 Gy. Target heart structures (red) and organs at risk such as the lungs (blue) and medulla (purple) are also shown. **(C)** Twelve to fourteen week old Wistar rats were cumulatively irradiated with 0.92, 6.9, or 27.6 Gy, administered in a fractionated scheme of 23 daily doses of 0.04, 0.3, or 1.2 Gy, respectively.

**Figure 2:**
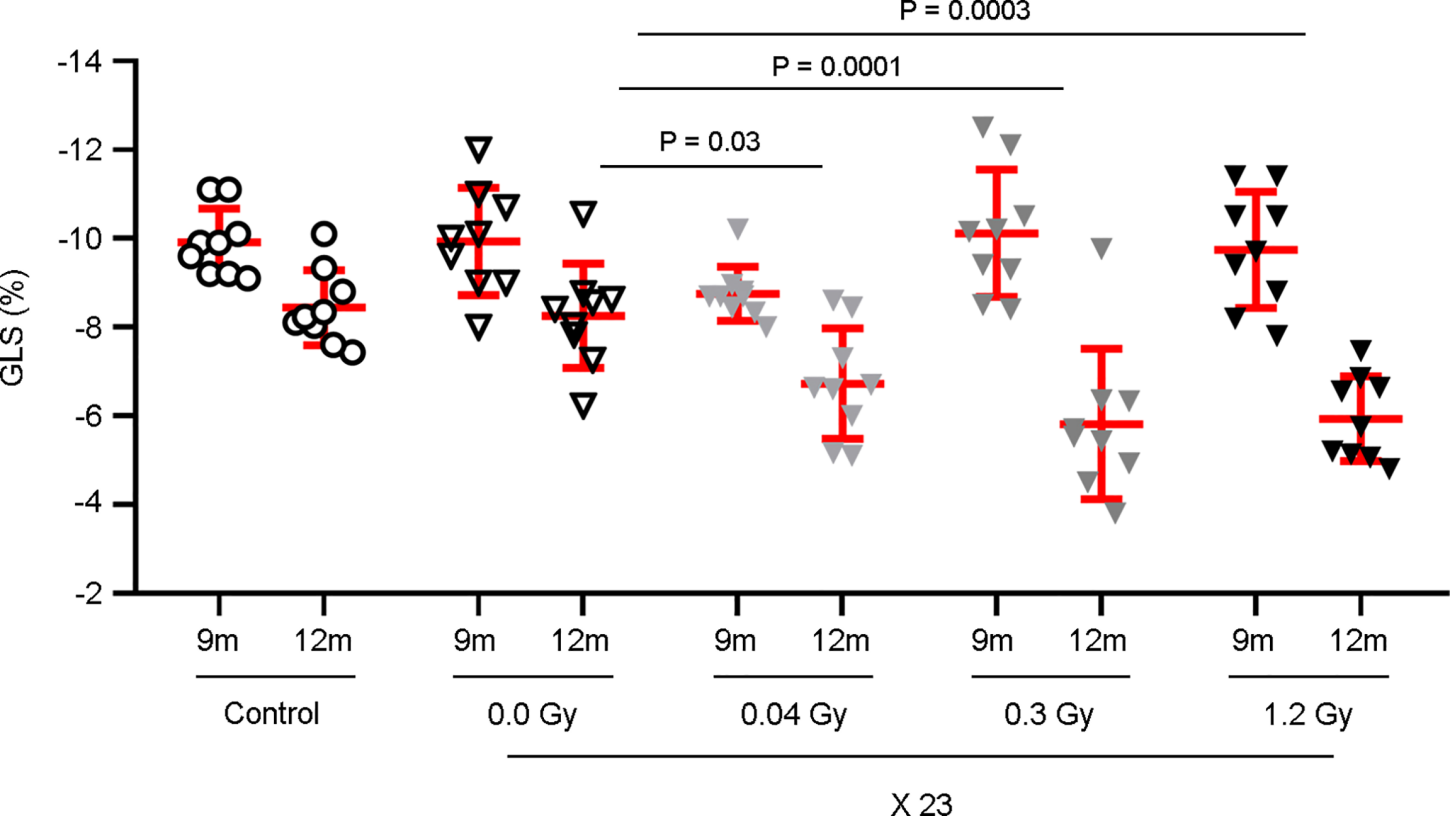
GLS is significantly decreased by the cumulative doses of 0.92, 6.9, and 27.6 Gy. Rat hearts were sham-irradiated or irradiated with 0.04, 0.3, and 1.2 Gy for 23 days. Rats, neither irradiated nor anesthetized, were used as controls. Echocardiography was performed at 9 and 12 months after the end of IR exposure. Measurements of the GLS show a significant decrease only at 12 months, with the daily doses of 0.04, 0.3, and 1.2 Gy. Individual data and mean ± SD (in red) are shown from n = 9 rats per group. Between-group changes were assessed by two-way repeated measurements ANOVA followed by a Bonferroni *post-hoc* test with a between-subject factor of irradiation, relative to the 0.0 Gy experimental group; significant *P-*values are displayed.

To evaluate the effect of radiotherapy on cardiac dysfunction, the hearts of adult Wistar rats were exposed to photon beams using a medical electron linear accelerator. Reference points were indicated with fiducial markers placed over the skin, followed by a CT simulation (**Figure 1A**) and radiotherapy planning (**Figure 1B**). The rat hearts were cumulatively exposed to 0.92, 6.9, or 27.6 Gy, covering a range from moderate to high doses of IR that are clinically relevant in a cardiac context for oncological patients who undergo radiotherapy. Importantly, these cumulative doses were administered in a fractionated scheme, meaning that the rat hearts were exposed daily to doses of 0.04, 0.3, or 1.2 Gy for 23 consecutive days (excluding weekends), to mimic the conventional radiotherapy plan (**Figure 1C**). A longitudinal study of cardiac structure and function by echocardiography was conducted (**Figures 2** , **S1–S4**). At 12 months after the end of radiotherapy, the chamber dimensions, LV systolic function, right ventricular function, and LV diastolic parameters were measured and no significant differences were observed (**Supplementary Figures S1–S4**), except for the relative percentage of GLS. According to our data, GLS presented a significant decrease of 18.5, 29.6, and 28.1% for the daily doses of 0.04, 0.3, and 1.2 Gy administered for 23 days, respectively, when compared to the sham-irradiated experimental group (**Figure 2**). At 9 months, no changes in GLS were observed for any cumulative dose (**Figure 2**).

It is also important to consider the decrease of GLS due to the aging process (8), observed both in control and sham experimental groups, between 9 and 12 months. Even though this effect is present in all experimental groups, GLS is clearly impaired by the additive effect of IR.

### Microvascular Density in the Cardiac Apex is Significantly Decreased by 27.6 Gy

**Figure 3:**
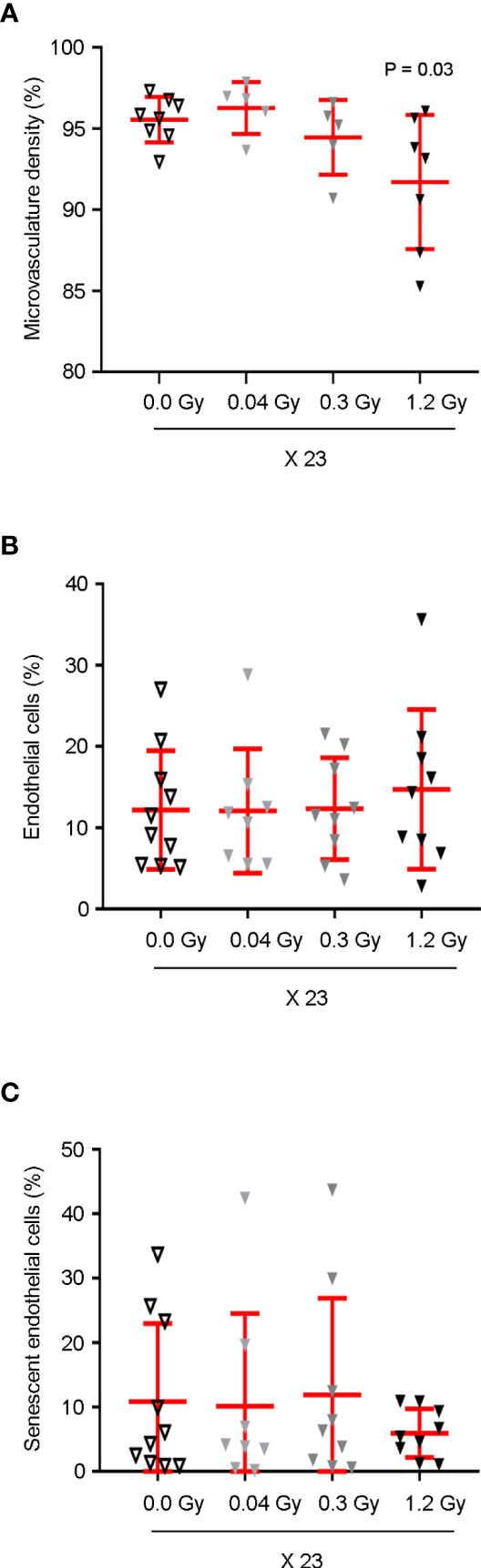
Cardiac microvasculature density is significantly decreased by the cumulative dose of 27.6 Gy. **(A)** Microvascular density in the cardiac apex of rats, sham-irradiated (n = 8) or irradiated with 23 daily fractions of 0.04 (n = 5), 0.3 (n = 5), or 1.2 Gy (n = 7), was evaluated by SEM, 12 months after IR exposure. Quantitative analysis of microvascular density is shown as a percentage of average capillary coverage relative to several successive ×100x magnification fields, demonstrating a significant decrease by the 1.2 Gy daily dose. One-way ANOVA combined with Bonferroni correction was used for statistical analysis. **(B, C)** Seven months after the end of IR exposure, **(B)** cardiac ECs were analyzed by flow cytometry based on cell percentage, in relation to total live single cells. **(C)** Cell senescence was assessed as the percentage of C12FDG positive ECs. **(B, C)** No significant differences were observed between groups n = 10 (0.0 Gy), n = 8 (0.04 Gy), n = 9 (0.3 Gy), and n = 9 (1.2 Gy). Kruskal–Wallis test with Dunn’s correction was used for statistical analysis. **(A–C)** Individual data and mean ± SD (in red) are shown. Significant *P-*values are displayed. EC, endothelial cell.

To evaluate the effect of the different cumulative doses of IR on the microvasculature density, four distinct regions of interest in the cardiac apex were evaluated by scanning electron microscopy (SEM). According to our data, the daily dose of 1.2 Gy administered for 23 days (27.6 Gy) significantly decreased the microvasculature density 12 months after the end of IR exposure (**Figure 3A**). According to these results, we decided to investigate the effects of the cumulative doses of 0.92, 6.9, or 27.6 Gy on the viability and senescence of endothelial cells (ECs), previously to the significant decrease in the microvasculature density in the cardiac apex. Therefore, the cardiac tissue was evaluated by flow cytometry seven months after the end of radiotherapy. No differences in the viability and senescence of ECs were observed between the different experimental groups (**Figures 3B, C**). Moreover, the percentage of cardiac fibroblasts and immune cells was also assessed, and no changes were found in the viability or senescence of fibroblasts, myeloid, B, or T cells (**Supplementary Figure S5**).

### The Gene Expression of Pro-Inflammatory, Pro-Fibrotic, and Oxidative Stress Markers are Modulated by 6.9 and/or 27.6 Gy

**Figure 4:**
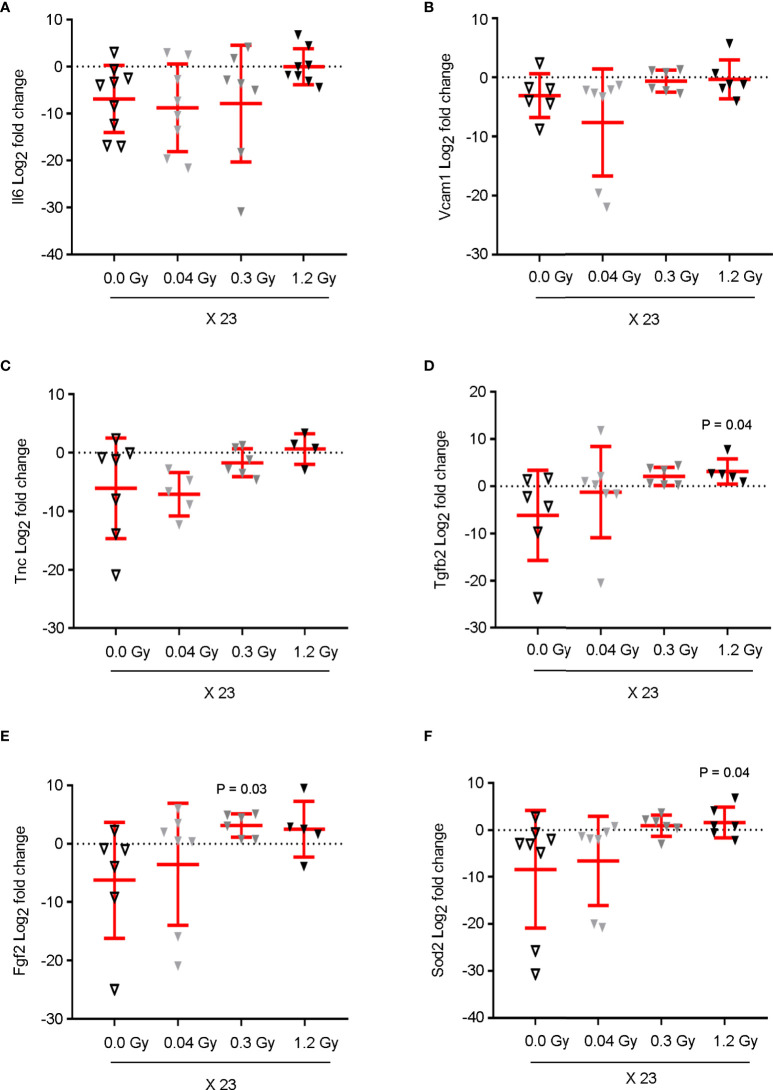
Cumulative doses of 6.9 and 27.6 Gy upregulate the mRNA expression of pro-inflammatory, pro-fibrotic, and oxidative stress markers in cardiac tissue, prior to myocardial strain dysfunction. The mRNA expression of **(A)** *Il6*, **(B)** *Vcam1*, **(C)** *Tnc*, **(D)** *Tgfb2*, **(E)** *Fgf2*, and **(F)** *Sod2* from the cardiac tissue of rats, sham-irradiated or irradiated with 0.04, 0.3, or 1.2 Gy for 23 daily fractions, was quantified by qRT-PCR. Cycle threshold values were normalized to 18S to obtain relative gene expression values. Data (mean ± SD) represents the Log_2_ of fold change in gene expression for each animal, in triplicate measurements relative to the fold change average of the sham-irradiated (0.0 Gy) group; n = 6–9 rats per group. Results show **(A)** an increasing trend of Il6 expression, exclusively in response to 1.2 Gy. **(B–F)** The levels of *Vcam1*, *Tnc*, *Tgfb2*, *Fgf2*, and *Sod2* present a significant augmentation or increasing trend in response to the daily doses of 0.3 and 1.2 Gy. A Kruskal–Wallis test with Dunn’s correction was used for statistical analysis; significant *P-*values are displayed. *Il6*, Interleukin6; *Vcam1*, Vascular cell adhesion molecule1; *Sod2*, Superoxide dismutase2; *Fgf2*, Fibroblast growth factor2; *Tgfb2*, Transforming growth factor beta2; *Tnc*, Tenascin C.

To study the putative effects in the cardiac tissue earlier to GLS impairment, several key targets involved in cardiac dysfunction were selected, mainly by their pro-inflammatory (IL6, VCAM1, TNC, CCL2, and E-Selectin), pro-fibrotic (FGF2, TGFβ2, TGFβ1, TNC, CCN2, COL1A1, FAP, Vimentin, THY1, and ACTA2), angiogenic (VCAM1, VEGF, and KDR), or anti-oxidation (SOD2) roles. Their gene expression was evaluated using quantitative real-time PCR (qRT-PCR) using heart tissue from rats that were sacrificed at 7 months after the end of radiotherapy. Our data show that, simultaneously, *Vcam1*, *Tnc*, *Tgfb2*, *Fgf2*, and *Sod2* have their expression levels increased (trend or significantly) in response to the daily doses of 0.3 and/or 1.2 Gy but not to 0.04 Gy (**Figure 4**). Moreover, the expression level of *Il6* showed a trend of augmentation, exclusively in response to the daily dose of 1.2 Gy. In contrast, the expression levels of *Ccl2*, *Sele*, *Tgfb1*, *Ccn2*, C*ol1a1*, *Fap*, *Vim*, *Thy1*, *Acta2*, *Vegfa*, and *Kdr* were not modulated by any dose of IR (**Supplementary Figure S6**). These results strongly suggest that at 7 months after the end of radiotherapy, the cardiac tissue is undergoing a modulation reflected by the change in gene expression, exclusively by the cumulative highest doses (6.9 and/or 27.6 Gy).

### Changes in the Cardiac Proteome Secondary to IR Exposure

According to the previous results, we decided to identify the proteins and key pathways that were modulated in the heart tissue 7 months after the end of IR exposure. The proteomics profile of the heart tissue (LV and atria) was evaluated by mass spectrometry (MS). Briefly, tissues were excised from equivalent heart regions from the different experimental groups and lysed. The extracted proteins were enzymatically digested with trypsin to peptides, followed by protein identification and quantification by high-resolution MS.

The reproducibility was very high, indicated by a median coefficient of variation lower than 3.2% for all conditions. Furthermore, the overall distribution of quantitative data across samples was comparable (**Supplementary Figure S7A**). Principal component analysis (PCA) revealed no separation for 0.04 Gy and then a gradually stronger separation of the samples according to the daily doses of 0.3 and 1.2 Gy (**Supplementary Figure S7B**). The PCA was in agreement with the overall statistical difference in quantitative data distribution, which dose-dependently increased when compared to the sham-irradiated group.

**Figure 5:**
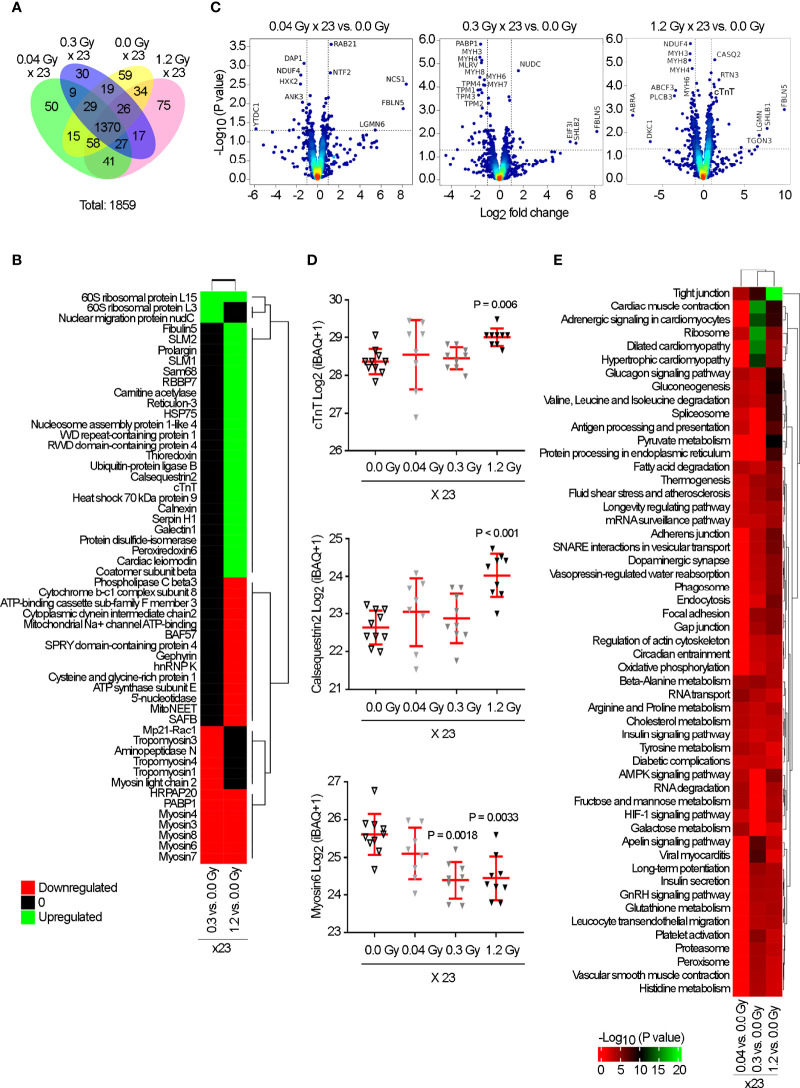
Proteomic analysis reveals a significant change in the expression of cTnT, Calsequestrin2, and Myosin6, in response to the cumulative doses of 0.92, 6.9, and 27.6 Gy. Rat hearts were sham-irradiated (n = 10) or irradiated with 0.04 (n = 8), 0.3 (n = 9), or 1.2 Gy (n = 9) for 23 daily fractions and analyzed by mass spectrometry at 7 months. **(A)** Venn diagram indicates the non- and overlapping number of proteins. **(B)** Heatmap represents the significantly up (green) and downregulated (red) proteins (adjusted *P*-value <0.05) from each group vs. sham-irradiated. **(C)** Volcano plots based on Log_2_ fold change against −Log_10_ (*P*-value) show the significantly up (upper right corner) and downregulated (upper left corner) proteins in each group vs. 0.0 Gy. **(D)** Quantitative Log_2_(iBAQ + 1) expression values show the significant upregulation of cTnT and Calsequestrin2 by 1.2 Gy, and downregulation of Myosin6 by 0.3 and 1.2 Gy. Individual data and mean ± SD (in red) are shown. A Kruskal–Wallis test with Dunn’s correction was used; significant *P-*values are displayed. **(E)** Heatmap of KEGG pathway enrichment analysis, for significantly dysregulated proteins, revealed “Tight junctions,” “Cardiac muscle contraction,” and “Adrenergic signaling in cardiomyocytes” as significantly enriched exclusively by 0.3 and 1.2 Gy. Color intensity indicates an enrichment score.

In total, 1,859 proteins were quantified from eight to ten biological replicates per experimental condition. Analysis of the Venn diagrams showed that 1,370 identified proteins were overlapping in all experimental groups (**Figure 5A**). Furthermore, p-values estimated using the limma package and corrected for multiple testing significantly identified 16 and 46 differentially expressed proteins in the cardiac tissue for the daily doses of 0.3 and 1.2 Gy, respectively, when compared with the sham-irradiated group (adjust *P* value <0.05; **Figure 5B**), while no proteins were detected for the daily dose of 0.04 Gy. Volcano plots for the comparison between sham-irradiated and the different daily doses administered for 23 days are provided (**Figure 5C**). Subsets of upregulated proteins and subsets of downregulated proteins were clearly separated from the general cloud of data points. From both analyses, several key proteins in cardiac muscle contraction were identified only in the higher cumulatively irradiated experimental groups, such as cardiac muscle Troponin T (cTnT), Calsequestrin2, and Myosin6 (**Figure 5D**). Therefore, we decided to evaluate the key pathways that could be significantly enriched in response to IR. KEGG analysis revealed functional diversity between the experimental groups (**Supplementary Figure S7C**). Interestingly, the KEGG pathways related to cardiac muscle “Tight junctions,” “Cardiac muscle contraction,” and “Adrenergic signaling in cardiomyocytes” were found significantly enriched in response to the cumulative doses of 6.9 and 27.6 Gy compared with the sham-irradiated experimental group, while no significant difference was found between 0.04 and 0.0 Gy (**Figure 5E**). Taken together, these data strongly support the putative contribution of cTnT, Calsequestrin2, and Myosin6 to cardiac dysfunction induced by IR.

### cTnT, Calsequestrin2, and Myosin6 Expression Levels are Modulated by 6.9 and 27.6 Gy

**Figure 6:**
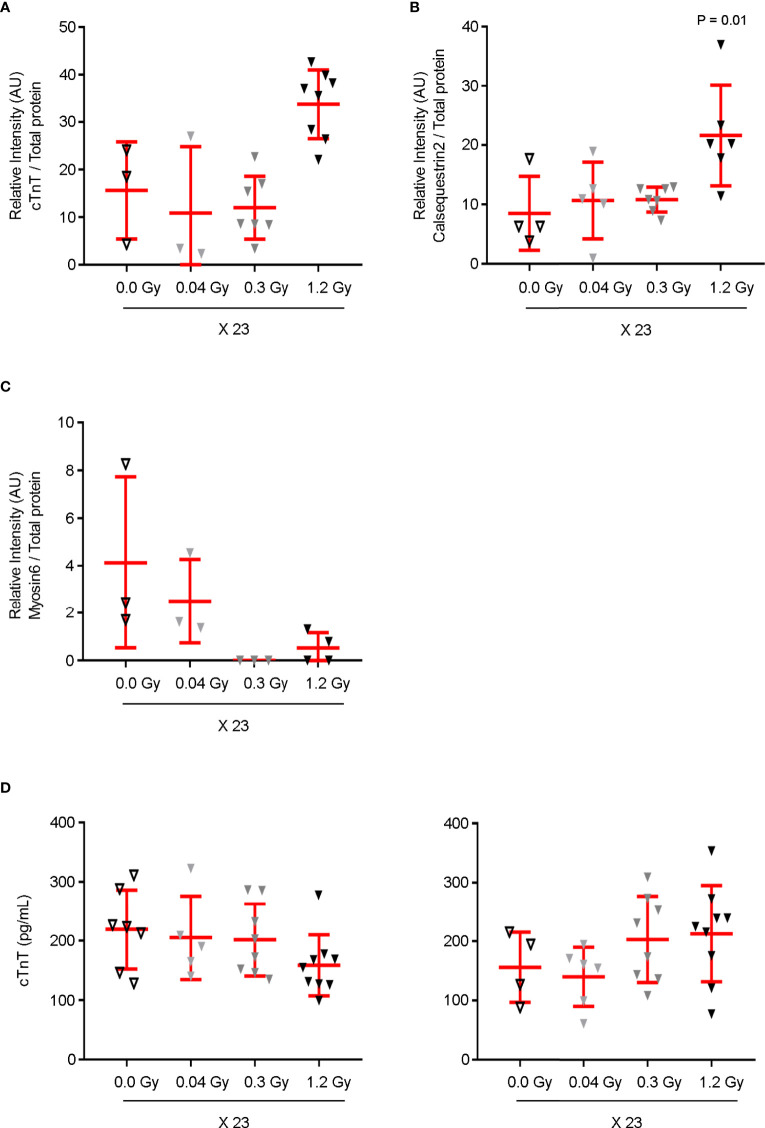
cTnT, Calsequestrin2, and Myosin6 expression levels are modulated in the cardiac tissue by the cumulative doses of 6.9 and/or 27.6 Gy. The cardiac tissue samples used in proteomics were analyzed by western-blot for **(A)** cTnT, **(B)** Calsequestrin2, and **(C)** Myosin6. Protein expression levels are shown as the relative densitometric intensity of the labeled proteins in relation to total protein in each lane. A Kruskal–Wallis test with Dunn’s correction was used for statistical analysis. Quantitative analysis shows **(A)** an increasing trend in the expression level of cTnT and **(B)** a significant augmentation of Calsequestrin2 in response to the daily dose of 1.2 Gy. **(C)** Myosin6 expression levels decrease by both daily doses of 0.3 and 1.2 Gy. **(D)** Plasma levels of circulating cTnT were assessed at 7 (left) and 12 months (right) after the end of IR exposure. Although no augmentation was detected at 7 months, an increasing trend was found for circulating cTnT in response to 0.3 and 1.2 Gy at 12 months. **(A–D)** Individual data and mean ± SD (in red) are shown for n = 3–9. cTnT, cardiac muscle troponin T.

The changes in expression levels of cTnT, Calsequestrin2, and Myosin6 by high cumulative doses of IR were validated in the same tissue samples used in proteomics by western-blot analysis, 7 months after the end of IR exposure. Compared to the sham experimental group, the cardiac tissue exposed to the daily dose of 1.2 Gy showed a trend of increase in cTnT protein expression and a significant upregulation of Calsequestrin2 levels (**Figures 6A, B**). Moreover, the levels of Myosin6 were clearly decreased by the daily doses of 0.3 Gy and 1.2 Gy (**Figure 6C**).

Circulating cTnT is an important biomarker of myocardial dysfunction (19). Its serum levels were assessed at 7 and 12 months (**Figure 6D**). As expected, no augmentation of circulating cTnT was observed at 7 months. In contrast, a decreasing trend was found exclusively for the highest daily dose (**Figure 6D**, left panel). Interestingly, later, at 12 months, an increasing trend was found for circulating cTnT in response to the daily doses of 0.3 and 1.2 Gy administered for 23 days (**Figure 6D**, right panel), suggesting that at this time point the highest cumulative doses promote subclinical cardiac dysfunction, corroborated by GLS impairment detection (**Figure 2**).

Taken together, these data strongly show that the cumulative doses of 6.9 Gy and 27.6 Gy modulate the expression of proteins in the cardiac tissue with a key role in cardiac muscle contraction before cardiac dysfunction.

### GLS Significantly Decreases in a Dose-Dependent Manner

Although we previously reported that the highest cumulative dose of 27.6 Gy induced a higher number of changes in the cardiac tissue compared with the cumulative dose of 6.9 Gy, the significant decrease in GLS level was similar for both cumulative doses. Therefore, no linear dose-response was observed for GLS at 12 months. This suggested that the differential effect between the highest doses was not reflected in the severity of GLS, at least at 12 months. Therefore, a similar experience was performed to evaluate the effect on GLS beyond 12 months.

**Figure 7:**
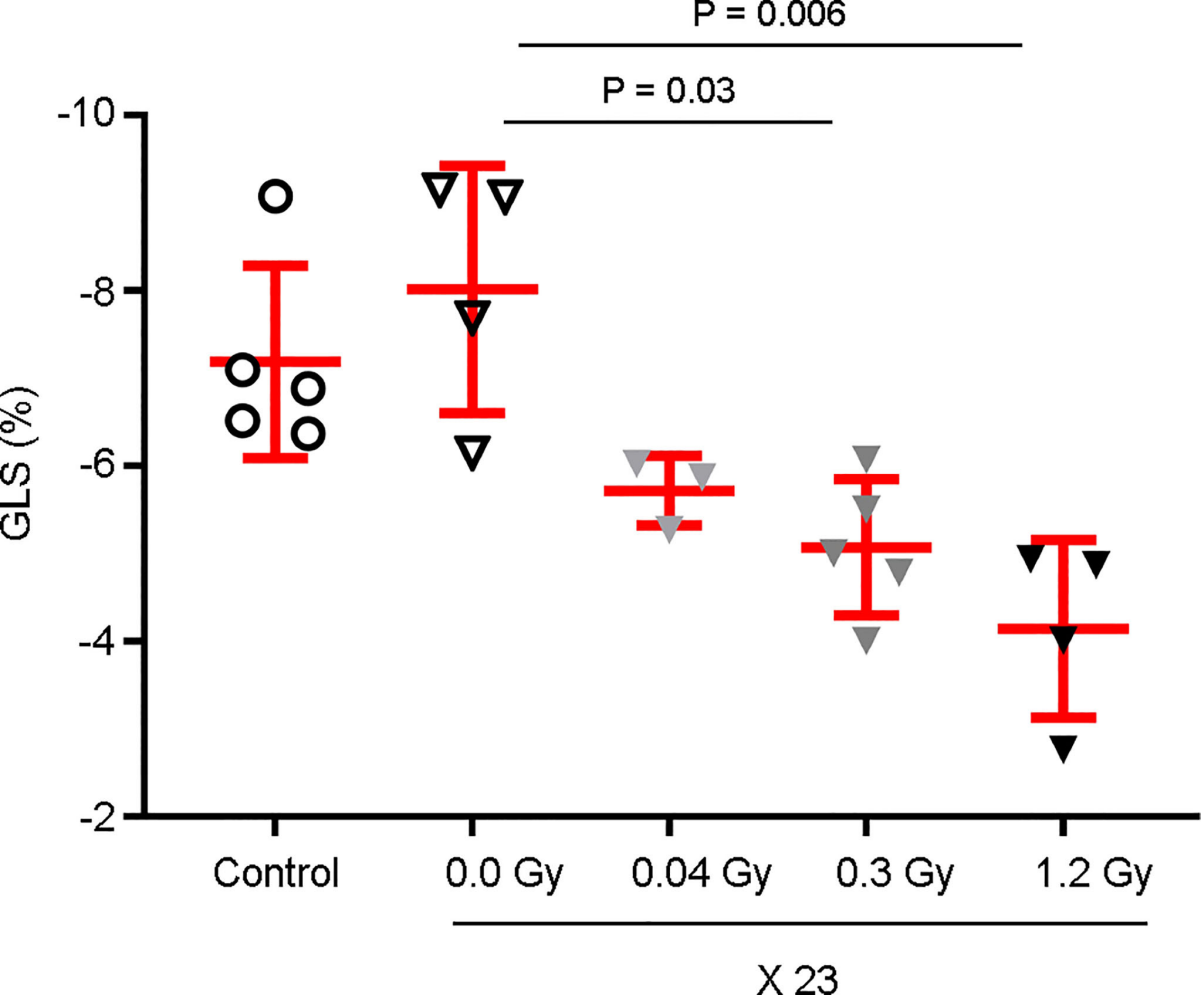
GLS is decreased in a dose-dependent manner 18 months after the end of IR exposure. Rat hearts were sham-irradiated (n = 4) or irradiated with 0.04 Gy (n = 3), 0.3 Gy (n = 5), and 1.2 Gy (n = 4) for 23 days. Rats neither irradiated nor anesthetized were used as controls (n = 5). Then, echocardiography was performed 18 months after the end of IR exposure and the GLS was measured. Observations show a decrease in a dose-dependent manner, with a significant difference associated with the 1.2 Gy daily dose. Individual data and mean ± SD (in red) are shown. A Kruskal–Wallis test with Dunn’s correction was used for statistical analysis; significant *P-*values are displayed.

According to our data, at 18 months after the end of IR exposure, the relative percentage of GLS showed a diminishing trend of 28.6% and a significant decrease of 36.7 and 48.3% for the daily doses of 0.04, 0.3, and 1.2 Gy administered for 23 days, respectively, when compared to the sham-irradiated experimental group (**Figure 7**). Overall, our data, besides validating those obtained at 12 months, showed that later, after 18 months, the GLS dose-dependently decreased.

## Discussion

An experimental model was established to evaluate the effect of IR *per se* on cardiac function and subclinical cardiac dysfunction, identifying the early molecular and cellular changes in the cardiac tissue upon IR exposure. Due to the life expectancy of rats, it was not our objective to assess the cardiotoxicity in this study. The cumulative doses of IR were administered in a fractionated manner, being clinically relevant for oncological patients who undergo thoracic radiotherapy. To understand the effects of IR exposure *per se*, no chemotherapeutic agents were combined with the IR. A longitudinal echocardiography assessment was performed to evaluate the effect of IR on cardiac function. According to our data, no structural or functional cardiac changes were detected until 18 months after exposure, except for the GLS. GLS is a marker for early LV subclinical dysfunction and is more sensitive and robust than other conventional LV functional parameters, such as LV ejection fraction (8). In human patients, a relative percentage reduction of >15% from baseline is considered abnormal (8). Our findings showed that daily doses of 0.04, 0.3, and 1.2 Gy, administered for 23 days, promote a significant decrease in GLS at 12 months after the end of IR exposure, unobserved earlier (at 9 months). Relative reductions of 18.5, 29.6, and 28.1% were shown for the cumulative doses of 0.92, 6.9, and 27.6 Gy, respectively, when compared to the control. Interestingly, later, at 18 months, GLS dose-dependently decreased. Relative reductions of 28.6, 36.7, and 48.3% were observed for the cumulative doses of 0.92, 6.9, and 27.6, respectively. Since it was reported that GLS *per se* decreases with age (8), we cannot exclude an additive effect of this factor overtime. However, its impact is similar in all experimental groups, strongly suggesting that GLS is differentially impaired according to the cumulative IR dose. It is crucial to understand the effects of radiotherapy *per se* on cardiotoxicity since radiation-associated cardiac disease occurs years or decades after chest radiotherapy and the diagnosis is challenging due to confounding effects from previous chemotherapy and/or other comorbidities, cardiac risk factors, and age (20). It was reported that a correlation between radiation dose and cardiac structures, particularly focused on coronary artery disease, as long-term outcomes (>10 years) after radiotherapy (21). Interestingly, a recent study involving chemotherapy-naive patients with breast cancer demonstrated impairment in myocardial strain in the short- to medium-term, which implies that subclinical cardiac dysfunction appears long before the onset of clinically significant cardiac events (22). Moreover, the segments that received the highest radiation dose also demonstrated the greatest myocardial strain impairment (22). This approach was not possible to consider in our study since all the cardiac structures were exposed to the same dose of radiation, given the small volume of the rat heart. However, different cumulative doses of IR were used, showing that GLS decreased in a dose-dependent manner, and highlighting for the first time the differential effects related to the dose in the cardiac tissue. Here, we found that when a significant decrease in GLS was detected, the microvasculature density in the cardiac apex was also significantly reduced; but, while GLS reduction was similar for the cumulative doses of 6.9 and 27.6 Gy, only the highest dose induced a significant reduction in the microvasculature density. These data strongly suggested the existence of other biological effects to justify GLS data, at 12 months. Therefore, we demonstrated that, effectively, the cardiac tissue was affected prior to the detection of any myocardial strain alterations, since although no change in GLS was detected at 9 months after the end of IR exposure, the expression of several genes and proteins related to inflammation, fibrosis, and cardiac muscle contraction were already modulated in the cardiac tissue at 7 months. Moreover, the highest cumulative dose of 27.6 Gy was correlated with a major number of targets that presented their gene or protein expression deregulated in the cardiac tissue, followed by the cumulative dose of 6.9 and finally 0.92 Gy. Seven months after the end of IR exposure, the gene expression of IL6, a pro-inflammatory cytokine, was significantly upregulated in the cardiac tissue in response exclusively to the cumulative dose of 27.6 Gy. The cardiac role of IL6 is complex and while its tissue expression was found to be associated with oxidative stress and apoptosis induction of cardiomyocytes, the increased circulating levels of the IL6 transcript and protein were reported as associated with a compensatory mechanism, leading to a cardioprotective action (23). Furthermore, other pro-inflammatory (VCAM1 and TNC) and pro-fibrotic (TNC, FGF2, and TGFβ2) targets are upregulated in response to the highest cumulative doses of 6.9 and 27.6 Gy, but their expression in response to the dose of 0.92 Gy was similar to the sham-irradiated group. In response to radiation, VCAM1 leads to the production of reactive oxygen species (ROS) by NADH oxidase and the activation of metalloproteinases to induce ventricular remodeling (24). TNC, a matricellular protein, is highly expressed under pathological conditions associated with cardiac inflammation and fibrosis (25). FGF2 was described as causative of cardiac disease and *Fgf2* knockout or overexpressing mice showed its role in inducing cardiac fibrosis by increasing fibroblast proliferation and interstitial collagen deposition (26). TGFβ2 is associated with the risk of radiation-induced fibrosis and myofibroblast-specific ablation of TGFβ2 effectively decreased fibrosis and conserved cardiac function (27). Finally, *Sod2* expression is also upregulated in response to 6.9 and 27.6 Gy. This could be justified by a compensatory mechanism against elevated oxidative stress and altered mitochondrial homeostasis, already described (28). The increased simultaneous transcription expression of these targets does not imply their corresponding protein expression but strongly suggests that, at 7 months, the cardiac tissue is undergoing a modulation compatible with later cardiac dysfunction. These data encouraged a proteomics analysis to identify the differential proteins and key functions that were modulated by the different cumulative doses of IR. As expected, the highest cumulative doses were the ones that showed a stronger separation of the samples in a dose-dependent manner relative to the control. The number of differentially expressed proteins in the cardiac tissue was dependent on the dose; the highest cumulative dose presented the greatest number of proteins. Interestingly, proteins with a crucial role in cardiac muscle contraction were identified, such as the cardiac muscle cTnT, Calsequestrin2, and Myosin6. The cTnT constitutes the cardiac troponin complex and is responsible for binding this complex to tropomyosin. Calsequestrin2 is a protein expressed in myocytes that is important in calcium storage and its release, essential to the excitation–contraction coupling (24). Our proteomics data, validated in the cardiac tissue, showed that cTnT and Calsequestrin expression levels were increased in response exclusively to the cumulative dose of 27.6 Gy. To our knowledge, this is the first study that reports an augmentation in the cardiac tissue of cTnT and Calsequestrin as an early effect, before the detection of any myocardial strain alterations, in response to high doses of IR. Accordingly, at 7 months, before GLS impairment detection, the levels of circulating cTnT were not augmented, and a decreasing trend was observed in response to the cumulative dose of 27.6 Gy. In contrast, at 12 months, when the GLS impairment was detected, its levels showed an increasing trend for 6.9 and 27.6 Gy, the same cumulative doses that presented the most significant decrease in GLS. Circulating cTnT is considered an important marker of myocardial damage (19). An early decrease in GLS and elevation in circulating cTnT levels have already been described as predictive of later cardiotoxicity in patients treated with chemotherapy (29). Concerning Caslsequestrin, even though the increase in its expression in the cardiac tissue was not reported so far, it is interesting to note that it was found that its cardiac-specific overexpression resulted in decreased myocardial contractility (30). Moreover, the expression of Myosin6 decreased in the cardiac tissue in response to 27.6 and 6.9 Gy. Myosin6 is the major protein comprising the cardiac muscle thick filament and functions in cardiac muscle contraction (31). Overall, this study demonstrates for the first time that, before myocardial strain impairment, IR induces differential deregulation of gene and protein expression in the cardiac tissue in a dose-dependent manner. The cardiac tissue proteins and genes affected by this process are altogether involved in cardiac dysfunction, particularly in muscle cardiac contraction, inflammation, fibrosis, and oxidative stress. Furthermore, the number of key targets and pathways affected by IR conditioned the severity of myocardial strain impairment. According to our findings, GLS decreased irreversibly, achieving worse values over time. We may speculate that, over time, other key targets and pathways present their expression/activation deregulated in response to the early modifications, contributing altogether to a dynamic cascade of modifications that will result later in a decrease in the microvasculature density, GLS impairment, and augmentation of the circulating cTnT. Concerning the microvascular density, it was not possible to find earlier (7 months) any evidence suggesting that the cumulative dose of 27.6 Gy promoted EC death or senescence. Both processes were described as being induced by high doses of IR, resulting in deleterious effects in cardiac micro- and macrovasculature (32). Importantly, microvascular dysfunction is a predictor of early LV remodeling, contributing to myocardial dysfunction (33). In our study, the assessment of the microvasculature density was only performed at 12 months when myocardial strain impairment was detected. This was a result of the complexity of the respective procedure, the impossibility of the heart being used simultaneously for any other analysis, and the obligation to sacrifice the animal. We consider that these facts constitute one limitation in our study as it was not possible to evaluate if the values decreased further after 12 months and if the other lower cumulative doses could induce similar effects later than 12 months. Another limitation of this study, is the entire irradiation of the heart and tumor absence, since we know that the human heart is not fully irradiated, and heart irradiation is a consequence of the tumor irradiation. However, if we can speculate on the advantages of these limitations, this study allowed us to determine the molecular targets and key pathways, differently changed by different cumulative doses of IR, in all the cardiac structures, in the absence of other confounding parameters that could be induced by the tumor itself.

**Figure 8:**
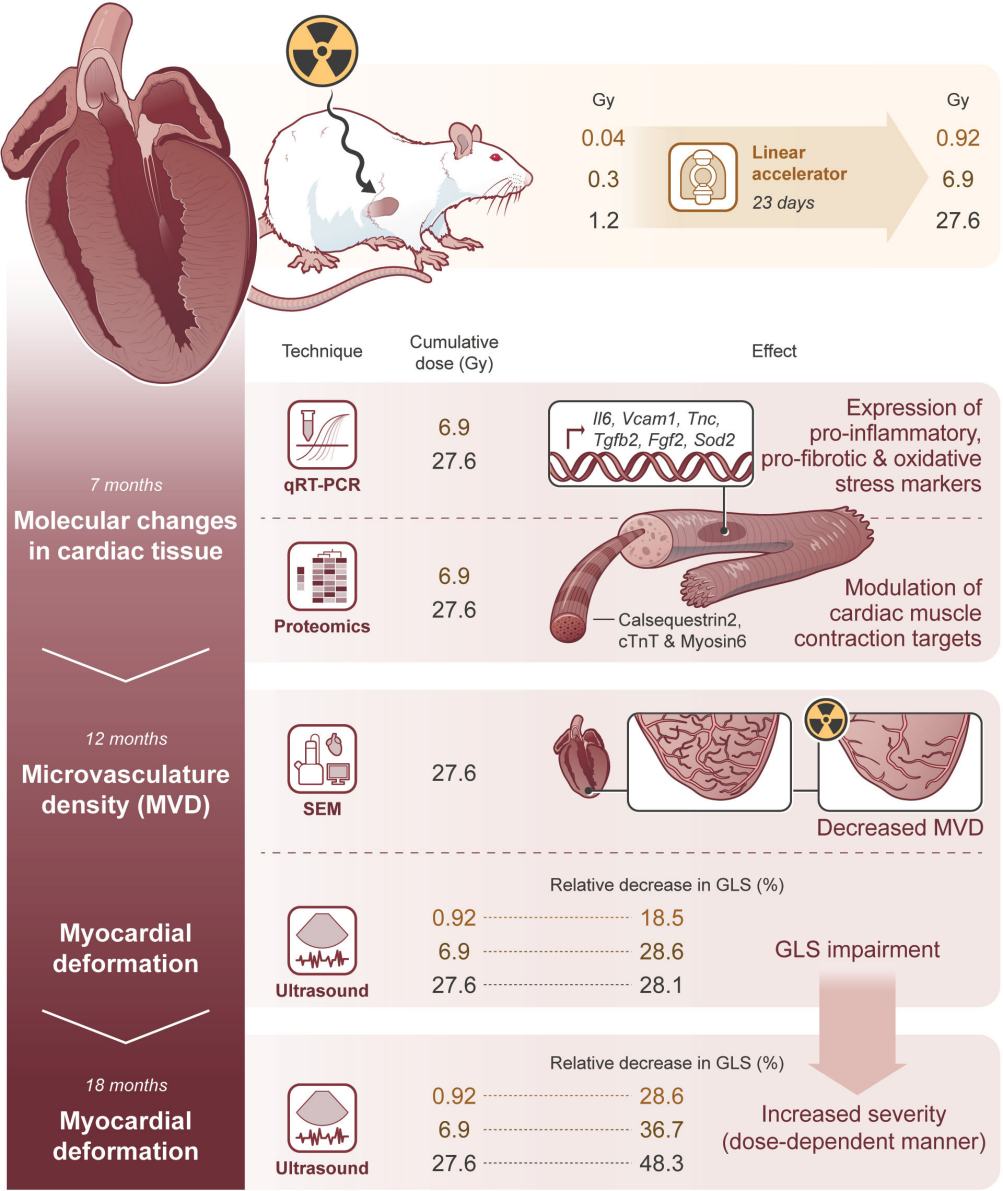
Molecular changes in cardiac tissue precede microvasculature density decrease and GLS impairment, affecting its severity in a dose-dependent manner. The illustrative figure summarizes the main conclusions of this study. Rat hearts were exposed to daily doses of 0.04, 0.3, and 1.2 Gy, achieving cumulative doses of 0.92, 6.9, and 27.6 Gy, respectively to evaluate the contribution of radiotherapy, *per se*, in late cardiotoxicity. At 7 months after the end of IR exposure, the cardiac tissue was modulated as demonstrated by the number/expression of several genes and proteins related to inflammation, fibrosis, and cardiac muscle contraction, differently expressed according to the cumulative dose. Importantly, the molecular changes in cardiac tissue precede microvascular density decrease and myocardial deformation, which are not observed until 12 months after the end of IR exposure. Later, at 18 months, the severity of GLS impairment is increased, demonstrating a myocardial deformation in a dose-dependent manner.

To conclude (**Figure 8**), this experimental model dose-dependently adds new knowledge by identifying key molecules and pathways in the cardiac tissue that contribute dynamically to cardiac changes after IR exposure. This process preceded the microvascular density decrease and myocardial deformation, unobserved until 12 months after the end of IR exposure. Later, after 18 months, the severity of GLS impairment is increased, demonstrating myocardial deformation in a dose-dependent manner, independent of the existence of other confounding factors.

This study opens new scenarios in the research of predictive markers of cardiac remodeling that should be further investigated, including the non-invasive quantification of diffuse myocardial fibrosis with cardiac magnetic resonance imaging. Furthermore, our study shows for the first time that daily low doses (0.04 Gy) achieving a cumulative dose lower than 1 Gy and daily moderate doses (0.3 and 1.2 Gy) corresponding to moderate (6.9 Gy) or high (27.6 Gy) cumulative doses, should be considered during heart exposure since their differential cardiac tissue effects will affect the severity of myocardial strain impairment and modulate the subclinical and subsequent clinical cardiac dysfunction. These data highlight the need for personalized clinical approaches and should raise red flags in radiation-based imaging and -guided therapeutic cardiac procedures, since there is no dose threshold below which no myocardial deformation impairment was detected.

## Data Availability Statement

The datasets presented in this study can be found in online repositories. The names of the repository/repositories and accession number(s) can be found in the article/**Supplementary Material**.

## Ethics Statement

The animal study was reviewed and approved by the Direção Geral de Alimentação e Veterinária (DGAV), the Portuguese competent authority for animal protection (license number 0421/000/000/2018).

## Author Contributions

SR and ASP performed echocardiography analysis. ASP, FR, IV, ATP, AM, MB, SF, RT, DC, HO, and RM performed the experimental work and analyzed the data. EP and ID planned and performed the irradiation experiments. SS conceived and designed the experiences in discussion with MF, FJP, DP, HO, and RM. SS wrote the manuscript. All authors contributed to the article and approved the submitted version.

## Funding

This work was supported by the European Community’s Horizon 2020 Program supported the MEDIRAD—Implications of Medical Low Dose Radiation Exposure granted by the Euratom Research and Training Program 2014-2014 under agreement No. 755523. The MS work was financed by the Portuguese Mass Spectrometry Network, integrated in the National Roadmap of Research Infrastructures of Strategic Relevance (ROTEIRO/0028/2013; LISBOA-01-0145-FEDER-022125). ARP and ATP were supported by a fellowship (SFRH/BD/121684/2016 and SFRH/BPD/123181/2016, respectively) and IV received a fellowship for Programmatic Funding (UIDP/00306/2020), all from Fundação para a Ciência e Tecnologia.

## Conflict of Interest

The authors declare that the research was conducted in the absence of any commercial or financial relationships that could be construed as a potential conflict of interest.

## Publisher’s Note

All claims expressed in this article are solely those of the authors and do not necessarily represent those of their affiliated organizations, or those of the publisher, the editors and the reviewers. Any product that may be evaluated in this article, or claim that may be made by its manufacturer, is not guaranteed or endorsed by the publisher.
