# Supplementary material for: Molecular Changes In Cardiac Tissue As A New Marker To Predict Cardiac Dysfunction Induced By Radiotherapy
Source: Front Oncol. 2022 Jul 26;12:945521. doi: 10.3389/fonc.2022.945521 (PMC9360508; doi:10.3389/fonc.2022.945521)
Supplement: Supplementary file 1 [file Presentation_1.pdf]

Supplementary Material

Data availability

The MS proteomics data underlying this article are available in ProteomeXchange Consortium via the PRIDE partner repository and can be accessed with the dataset identifier PXD033194 and 10.6019/PXD033194.

Supplementary figures

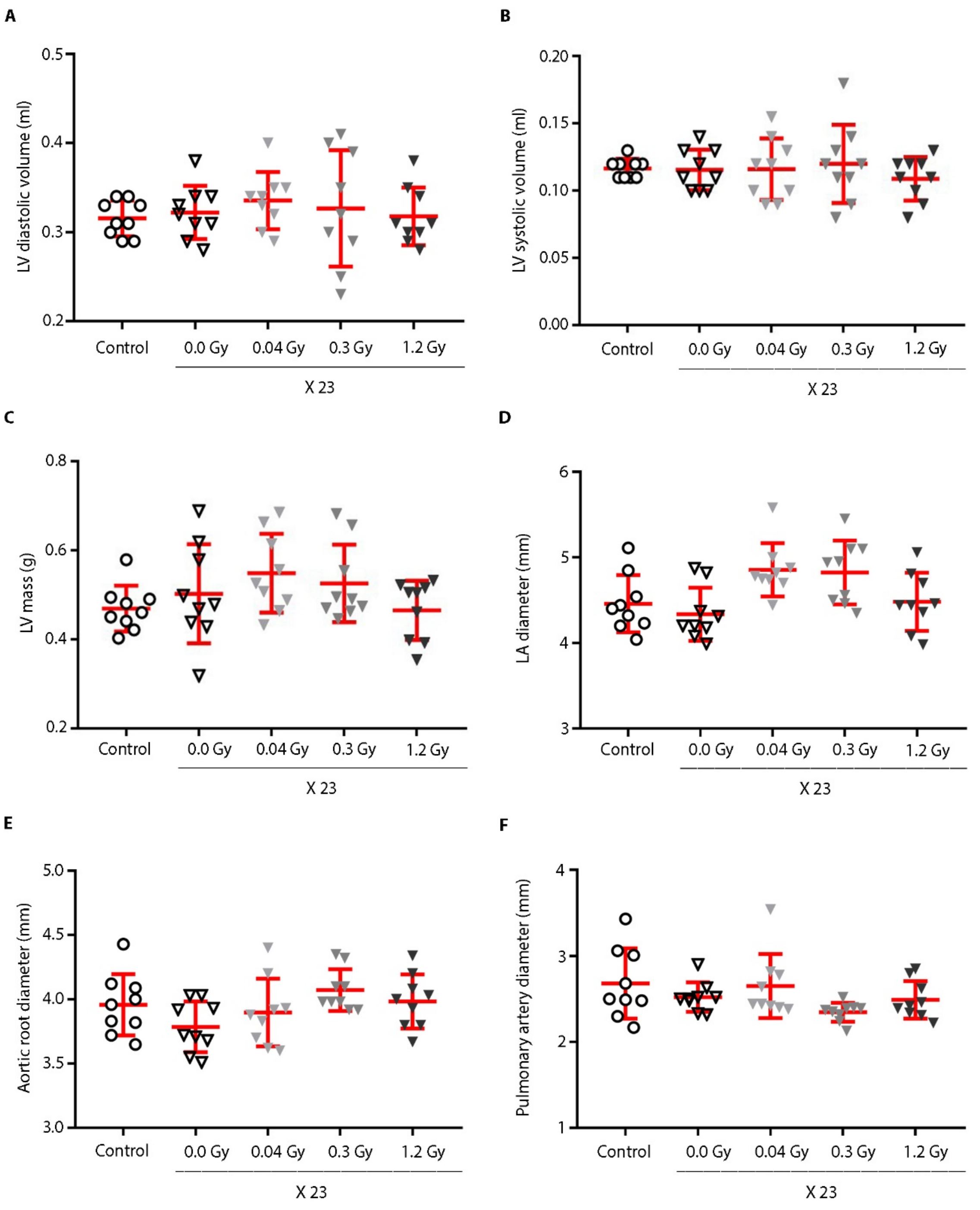

Supplementary Figure S1. Cardiac chambers are not affected by the exposure to cumulative doses of IR, at 12 months. Rat's hearts were sham-irradiated or irradiated with 0.04 Gy, 0.3 Gy and 1.2 Gy for 23 days. Rats neither irradiated nor anesthetized were used as control. Echocardiography was performed 12 months after IR exposure and chamber dimensions measured. **(A)** Left ventricle (LV) diastolic volume; **(B)** LV systolic volume; **(C)** LV mass; (D) Left atrium (LA) diameter; (E) Aortic root diameter; (F) Pulmonary artery diameter. No differences are observed between the different

experimental groups. Individual data and mean ± SD (in red) for n=9 rats are shown. Kruskal–Wallis test with Dunn's correction was used for statistical analysis.

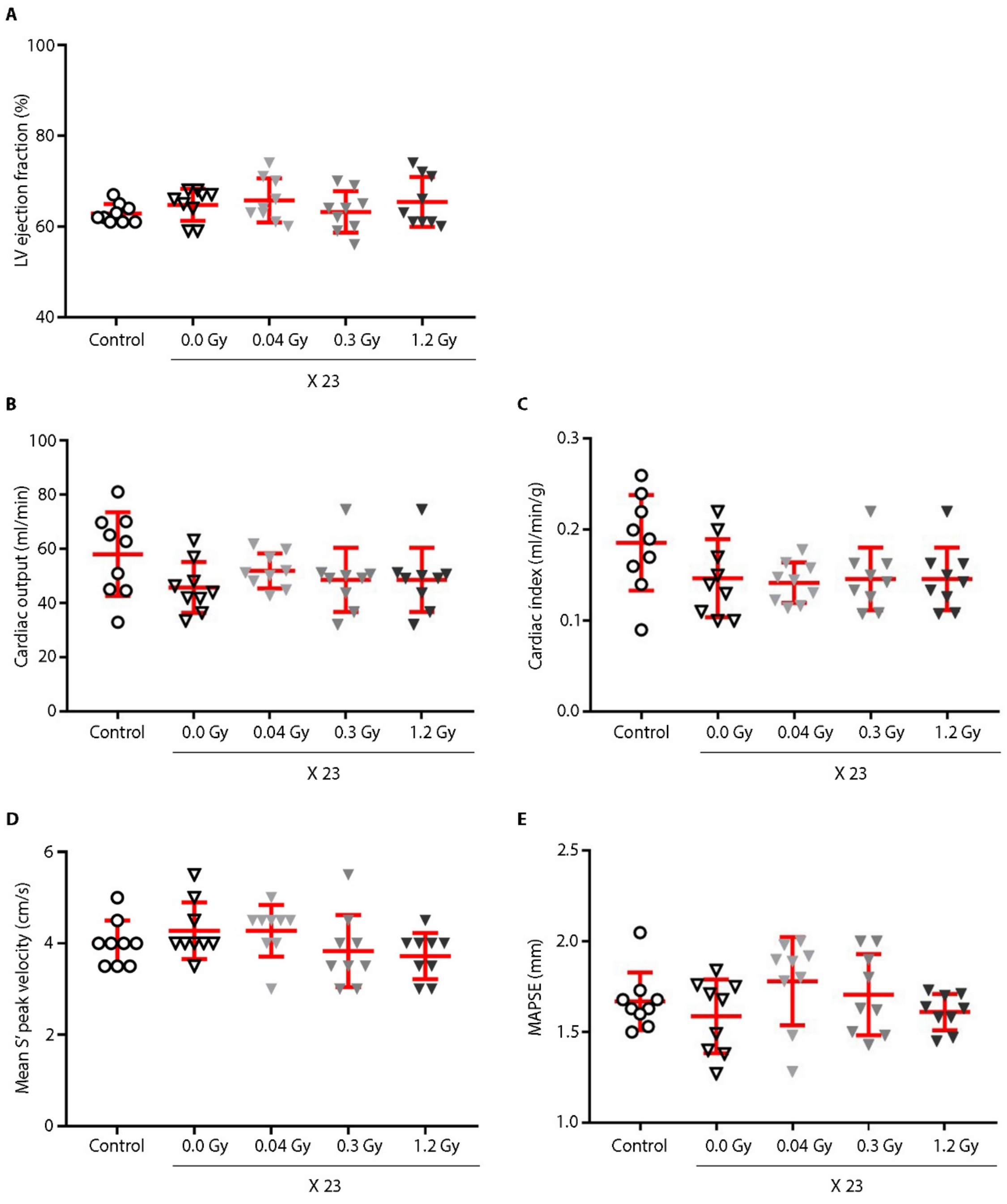

Supplementary Figure S2. Left ventricular systolic function is not changed by the exposure to cumulative doses of IR, at 12 months. Rat's hearts were sham-irradiated or irradiated with 0.04 Gy, 0.3 Gy and 1.2 Gy for 23 days. Rats neither irradiated nor anesthetized were used as control. Echocardiography was performed 12 months after IR exposure and left ventricular systolic function measured. **(A)** Left ventricular (LV) ejection fraction; **(B)** Cardiac output; **(C)** Cardiac index; **(D)** Mean S'-wave peak velocity of spectral/pulsed tissue Doppler in mitral annulus at septal and lateral wall; **(E)** Mitral annular plane systolic excursion (MAPSE). No differences are observed between the different experimental groups. Individual data and mean ± SD (in red) for n=9 rats are shown. Kruskal–Wallis test with Dunn's correction was used for statistical analysis.

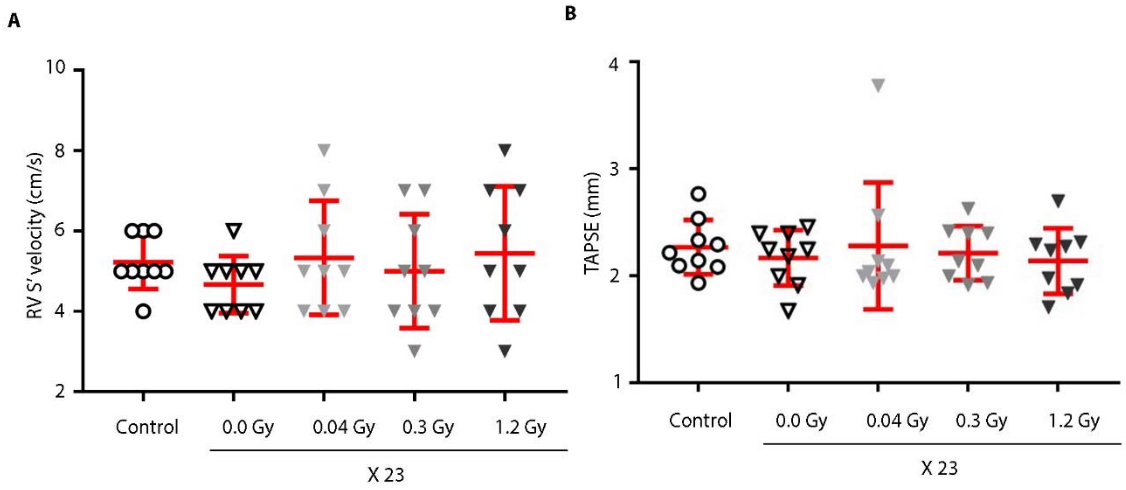

Supplementary Figure S3. Right ventricular function is not changed by the exposure to cumulative doses of IR, at 12 months. Rat's hearts were sham-irradiated or irradiated with 0.04 Gy, 0.3 Gy and 1.2 Gy for 23 days. Rats neither irradiated nor anesthetized were used as control. Echocardiography was performed 12 months after IR exposure and the right ventricular (RV) function measured. **(A)** S' wave at RV lateral wall, with pulsed doppler sample volume placed at tricuspid annulus (RV S' velocity); **(B)** Tricuspid annular plane systolic excursion (TAPSE). No differences are observed between the different experimental groups. Individual data and mean ± SD (in red) for n=9 rats are shown. Kruskal–Wallis test with Dunn's correction was used for statistical analysis.

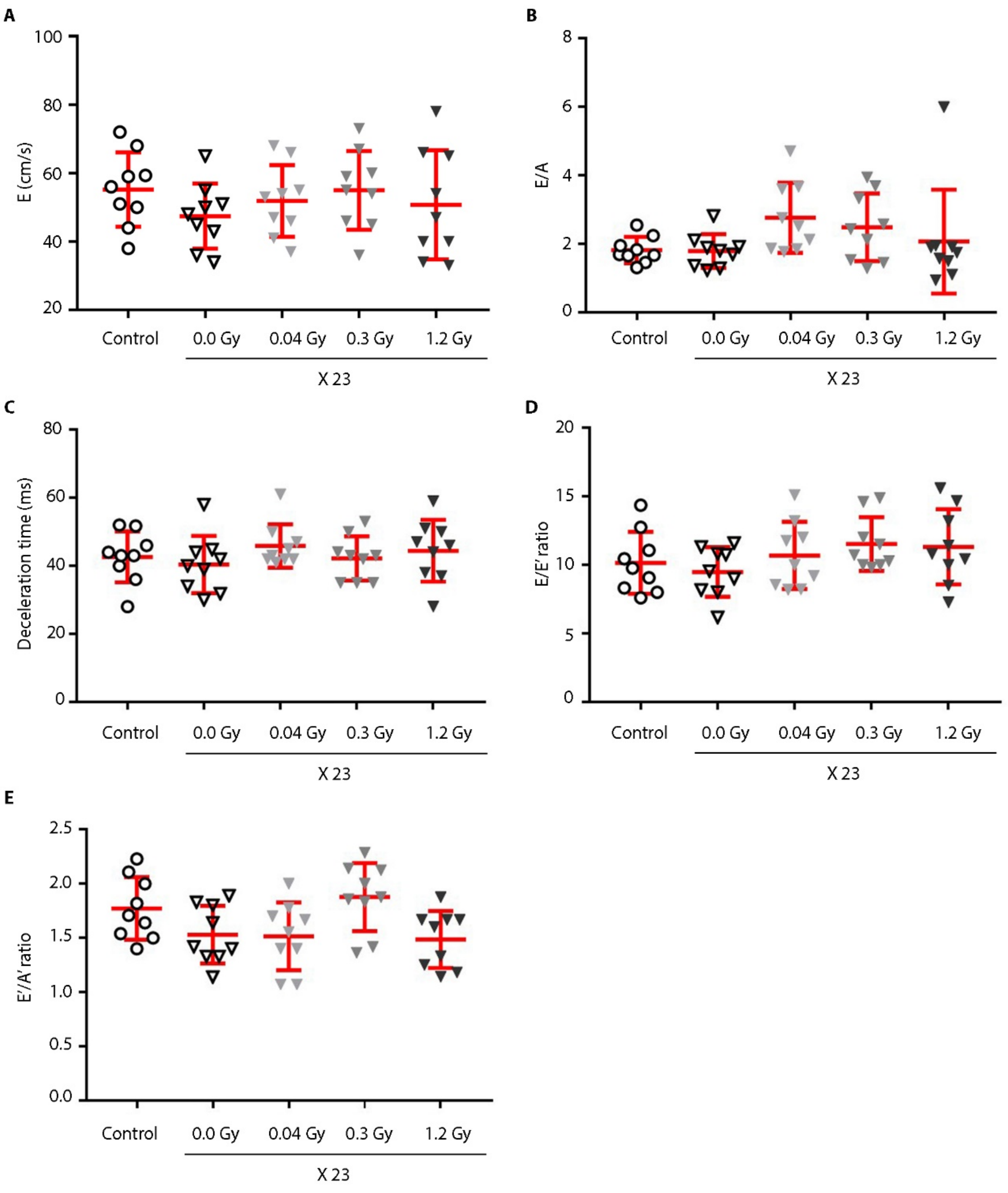

Supplementary Figure S4. Left ventricular diastolic parameters are not affected by the exposure to cumulative doses of IR, at 12 months. Rat's hearts were sham-irradiated or irradiated with 0.04 Gy, 0.3 Gy and 1.2 Gy for 23 days. Rats neither irradiated nor anesthetized were used as control. Echocardiography was performed 12 months after IR exposure and left ventricular diastolic parameters measured. **(A)** Peak E-wave velocity (E); **(B)** Ratio between E and A waves peak velocities (E/A ratio); **(C)** E-wave deceleration time; **(D)** Ratio between early mitral inflow velocity and mitral annular early diastolic velocity (E/E'); **(E)** Ratio between early and late diastolic velocity (E'/A'). No differences are observed between the different experimental groups. Individual data and mean ± SD (in red) for n=9 rats are shown. Kruskal–Wallis test with Dunn's correction was used for statistical analysis.

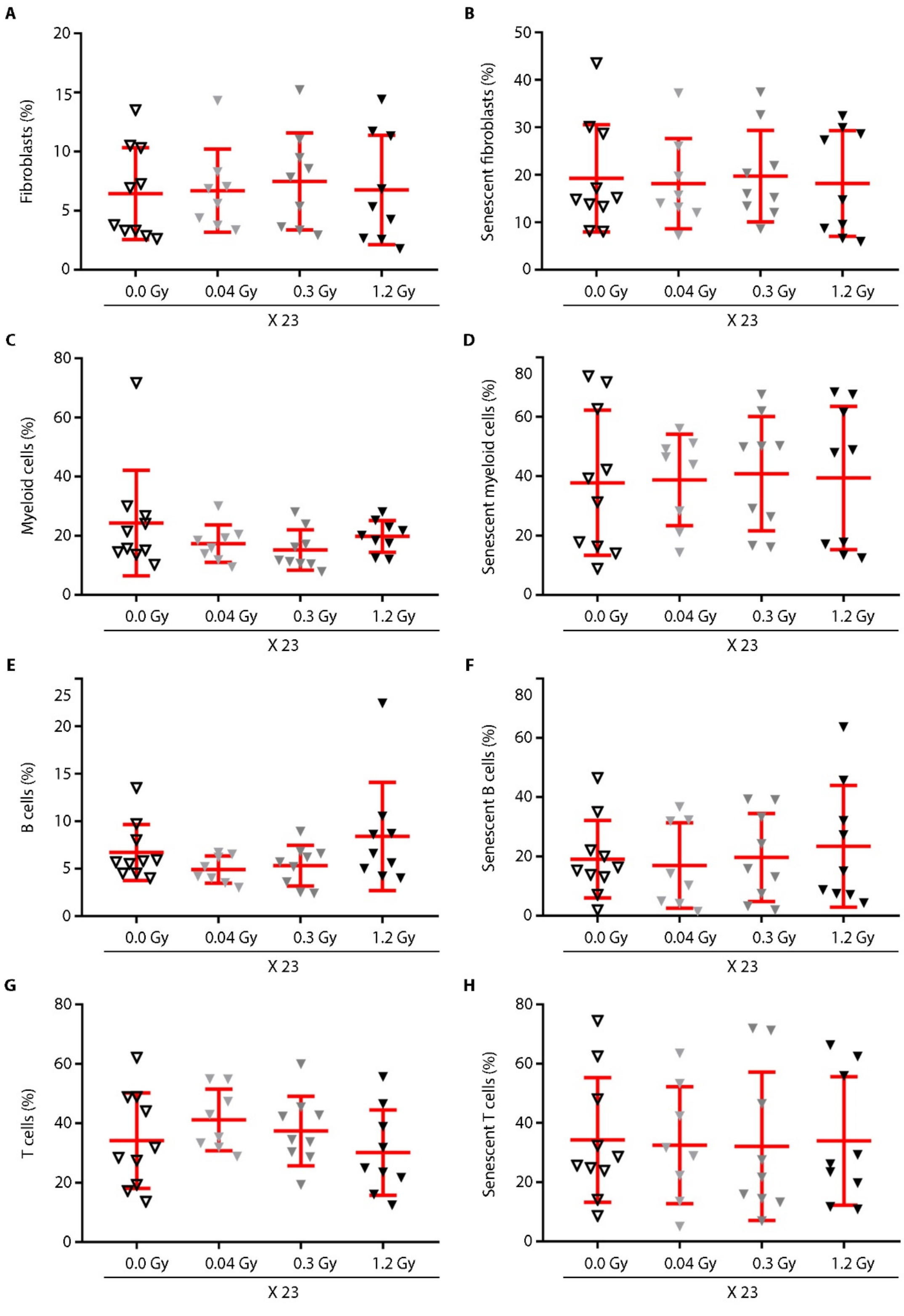

Supplementary Figure S5. The viability and senescence of cardiac fibroblast and immune cells are not affected by the exposure to cumulative doses of IR, at 7 months post-IR exposure. The cell populations of the cardiac tissue of rats, sham-irradiated (n=10) or irradiated with 23 daily fractions of 0.04 (n=8), 0.3 (n=9) or 1.2 Gy (n=9), was characterized by flow cytometry. **(A)** Cardiac fibroblasts were analysed based on cell percentage, in relation to total live single cells. **(C)** Myeloid, **(E)** B and **(G)** T cells were analysed based on cell percentage in relation to total CD45 positive cells, since the

number of immune cells was too low to be normalized by total live single cells. Cell senescence was assessed by analysing the percentage of fibroblasts, myeloid, B and T cells that were C12FDG positive (**B**, **D**, **F**, and **H**, respectively). No differences are observed between the different experimental groups. Individual data and mean ± SD (in red) are shown. Kruskal–Wallis test with Dunn's correction was used for statistical analysis.

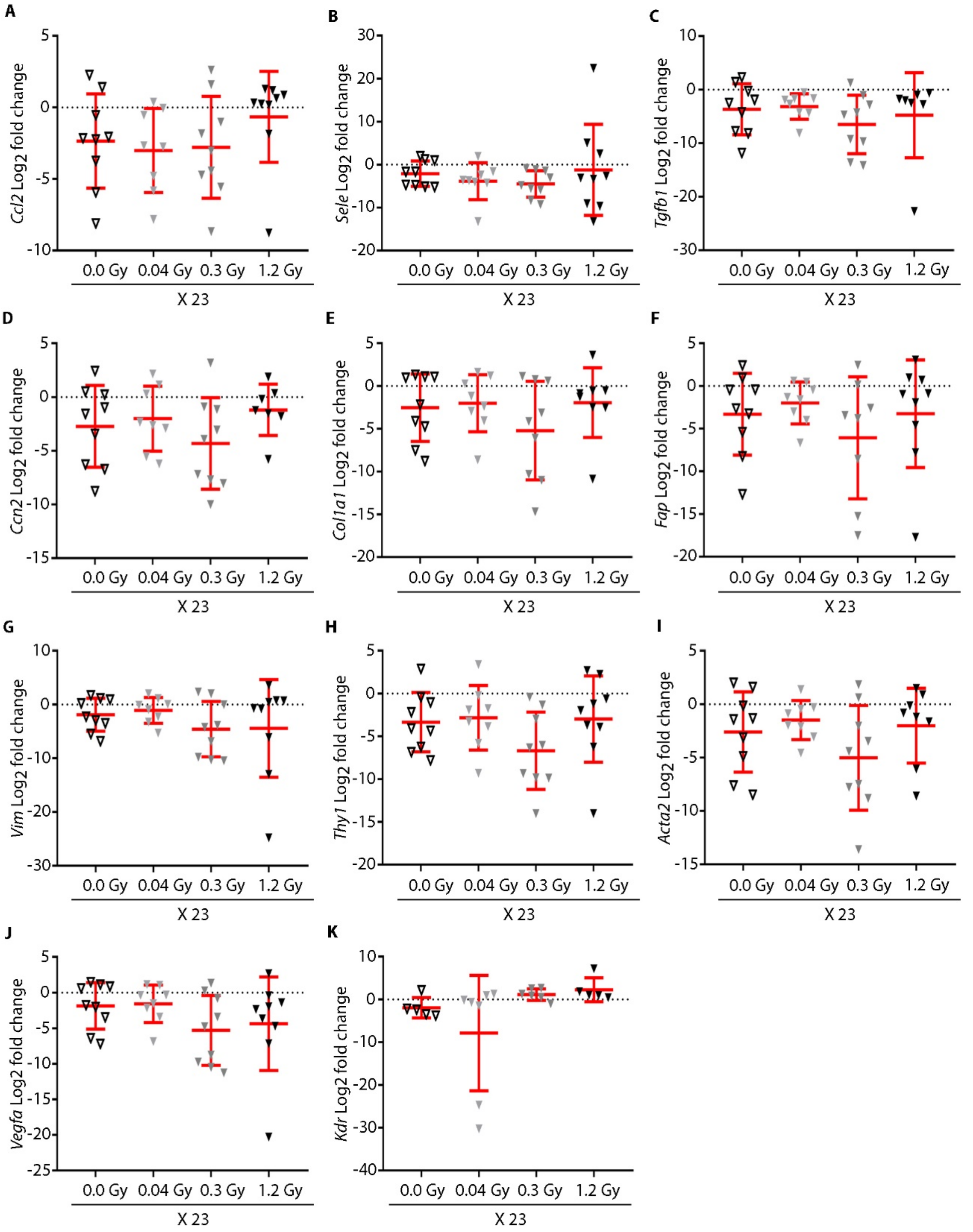

Supplementary Figure S6. mRNA expression levels of certain pro-inflammatory, pro-fibrotic and angiogenic markers are not modulated in cardiac tissue by the exposure to cumulative doses of IR, at 7 months post-IR exposure. The mRNA expression of **(A)** *Ccl2*, **(B)** *Sele*, **(C)** *Tgfb1*, **(D)** *Ccn2*, **(E)** *Col1a1*, **(F)** *Fap*, **(G)** *Vim*, **(H)** *Thy1*, **(I)** *Acta2*, **(J)** *Vegfa*, **(K)** *Kdr* from cardiac tissue of rats, sham-irradiated or irradiated with 0.04, 0.3 or 1.2 Gy for 23 daily fractions, was quantified by qRT-PCR. Values were normalized to 18S to obtain relative gene expression values. Data (mean ± SD) represent the Log$_2$ of fold change in gene expression for each animal, in triplicate measurements relative to the fold change average of the sham-irradiated (0.0 Gy) group; n=5-9 rats per group. Kruskal–Wallis test with Dunn's correction was used for statistical analysis. No significant differences are found. *Ccl2*, C-C motif chemokine 2 gene; *Sele*,

E-selectin gene; *Tgfb1*, Transforming growth factor beta1; *Ccn2*, CCN family member2; *Col1a1*, Collagen alpha-1(I) chain; *Fap*, Fibroblast activation protein gene; *Vim*, Vimentin gene; *Thy1*, Thy1 membrane glycoprotein; *Acta2*, Alpha-actin2; *Vegfa*, Vascular endothelial growth factor A; *Kdr,* Receptor protein-tyrosine kinase.

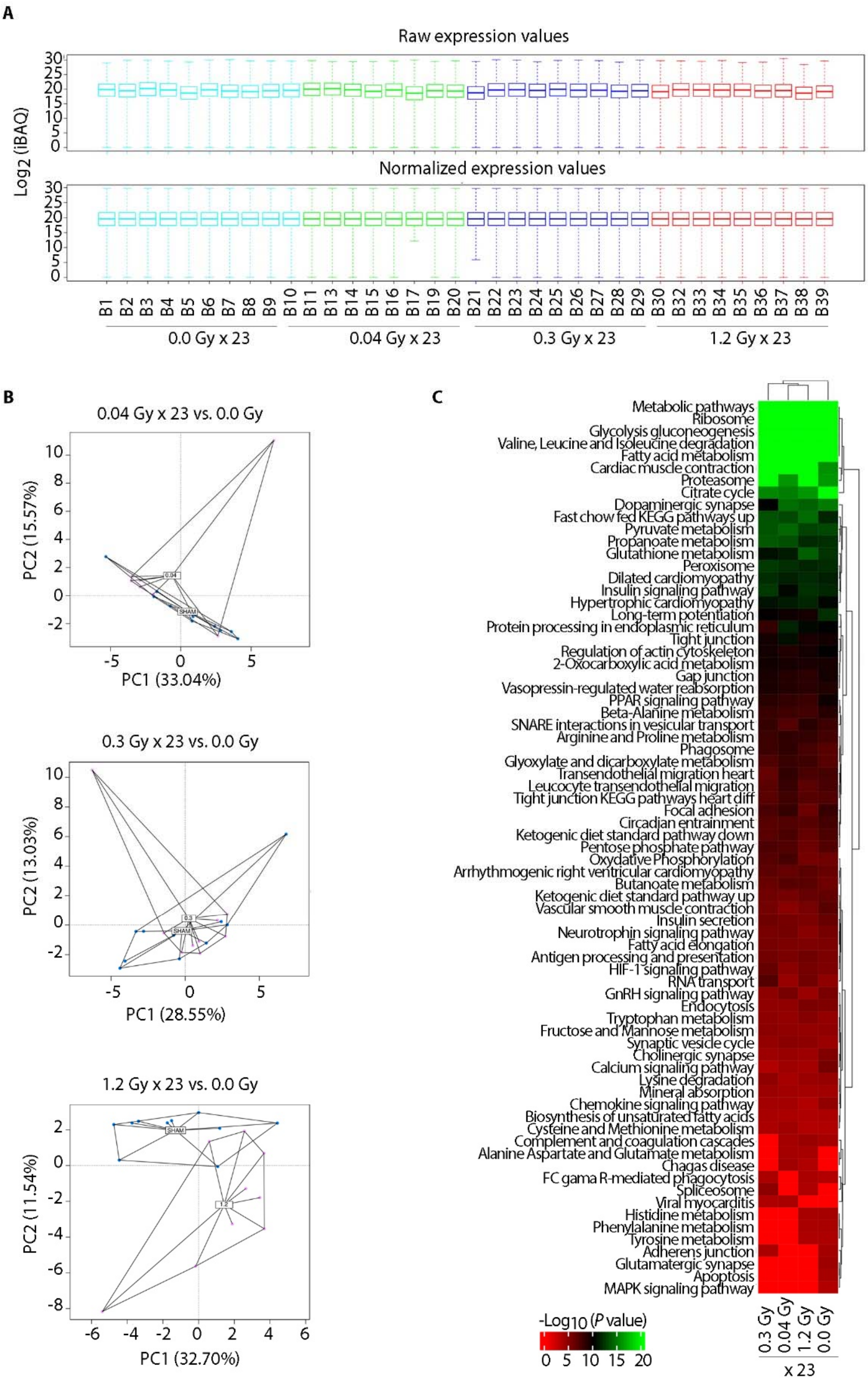

Supplementary Figure S7. Cardiac tissue proteome is modulated in a dose-dependent manner, 7 months after IR exposure. Rat's hearts were sham-irradiated (n=10) or irradiated with 0.04 (n=8), 0.3 (n=9) or 1.2 Gy (n=9) for 23 daily fractions and analysed by mass spectrometry, 7 months post-IR exposure. **(A)** Distribution of quantitative Log_2(iBAQ) expression values in each sample (B1-B10, B11-B20, B21-B29, B30-B39

from 0.0, 0.04, 0.3 and 1.2 Gy, respectively) is represented by a boxplot, for raw (up) and normalized (down) values. Overall distribution of quantitative data across samples from distinct experimental groups was comparable. **(B)** Principal component analysis of proteomic data in two-dimensional scatterplots of two principal components (PC1 and PC2), comparing 0.0 Gy with the 0.04 (up), 0.3 (middle) or 1.2 Gy (down) groups. Scatter plots indicate a significant variance in the proteomic profile in response to daily doses of 0.3 and 1.2 Gy, with a stronger separation observed for the highest dose (down). **(C)** Heatmap for enriched KEGG pathways. Colour range represents $-\text{Log}_{10}$ of enrichment *P*-value for the identified proteins in each experimental group. Functional diversity between groups is observed, with samples clustering according to the daily IR dose.
